# Multi-Timescale Memory Dynamics Extend Task Repertoire in a Reinforcement Learning Network With Attention-Gated Memory

**DOI:** 10.3389/fncom.2018.00050

**Published:** 2018-07-12

**Authors:** Marco Martinolli, Wulfram Gerstner, Aditya Gilra

**Affiliations:** School of Computer and Communication Sciences, School of Life Sciences, Brain-Mind Institute, École Polytechnique Fédérale de Lausanne, Lausanne, Switzerland

**Keywords:** reinforcement learning, memory, attention, synaptic plasticity, eligibility trace, synaptic tagging

## Abstract

The interplay of reinforcement learning and memory is at the core of several recent neural network models, such as the Attention-Gated MEmory Tagging (AuGMEnT) model. While successful at various animal learning tasks, we find that the AuGMEnT network is unable to cope with some hierarchical tasks, where higher-level stimuli have to be maintained over a long time, while lower-level stimuli need to be remembered and forgotten over a shorter timescale. To overcome this limitation, we introduce a hybrid AuGMEnT, with leaky (or short-timescale) and non-leaky (or long-timescale) memory units, that allows the exchange of low-level information while maintaining high-level one. We test the performance of the hybrid AuGMEnT network on two cognitive reference tasks, sequence prediction and 12AX.

## 1. Introduction

Memory spans various timescales and plays a crucial role in human and animal learning (Tetzlaff et al., 2012). In cognitive neuroscience, the memory system that enables manipulation and storage of information over a period of a few seconds is called Working Memory (WM), and is correlated with activity in prefrontal cortex (PFC) and basal ganglia (BG) (Mink, 1996; Frank et al., 2001). In computational neuroscience, there are not only several standalone models of WM dynamics (Samsonovich and McNaughton, 1997; Compte et al., 2000; Barak and Tsodyks, 2014), but also supervised and reinforcement learning models augmented by working memory (Graves et al., 2014, 2016; Alexander and Brown, 2015; Rombouts et al., 2015; Santoro et al., 2016).

Memory mechanisms can be implemented by enriching a subset of artificial neurons with slow time constants and gating mechanisms (Hochreiter and Schmidhuber, 1997; Gers et al., 2000; Cho et al., 2014). More recent memory-augmented neural network models like the Neural Turing Machine (Graves et al., 2014) and the Differentiable Neural Computer (Graves et al., 2016), employ an addressable memory matrix that works as a repository of past experiences and a neural controller that is able to store and retrieve information from the external memory to improve its learning performance.

Here, we study and extend the Attention-Gated MEmory Tagging model or AuGMEnT (Rombouts et al., 2015). AuGMEnT is trained with a Reinforcement Learning (RL) scheme, where learning is based on a reward signal that is received after each action selection. The representation of stimuli is accumulated in the memory states and the memory is reset at the end of each trial (see Methods). The main advantage of the AuGMEnT network for the computational neuroscience community resides in the biological plausibility of its learning algorithm.

Notably, the AuGMEnT network uses a memory-augmented version of a biologically plausible learning rule (Roelfsema and van Ooyen, 2005) mimicking backpropagation (BP). Learning is the result of the joint action of two factors, neuromodulation and attentional feedback, both influencing synaptic plasticity. The former is a global reward-related signal that is released homogeneously across the network to inform each synapse of the reward prediction error after response selection (Schultz et al., 1993, 1997; Waelti et al., 2001). Neuromodulators such as dopamine influence synaptic plasticity (Yagishita et al., 2014; Brzosko et al., 2015, 2017; He et al., 2015; Frémaux and Gerstner, 2016). The novelty of AuGMEnT compared to three-factor rules (Xie and Seung, 2004; Legenstein et al., 2008; Vasilaki et al., 2009; Frémaux and Gerstner, 2016) is to add an attentional feedback system in order to keep track of the synaptic connections that cooperated for the selection of the winning action and overcome the so-called structural credit assignment problem (Roelfsema and van Ooyen, 2005; Rombouts et al., 2015). AuGMEnT includes a memory system, where units accumulate activity across several stimuli in order to solve temporal credit assignment tasks involving delayed reward delivery (Sutton, 1984; Okano et al., 2000). The attentional feedback mechanism in AuGMEnT works with: (a) synaptic eligibility traces that decay slowly over time, and (b) non-decaying neuronal traces that store the history of stimuli presented to the network up to the current time (Rombouts et al., 2015). The AuGMEnT network solves the Saccade-AntiSaccade task (Rombouts et al., 2015), which is equivalent to a temporal XOR task (Abbott et al., 2016) (see Supplementary Material A).

**Figure 1:**
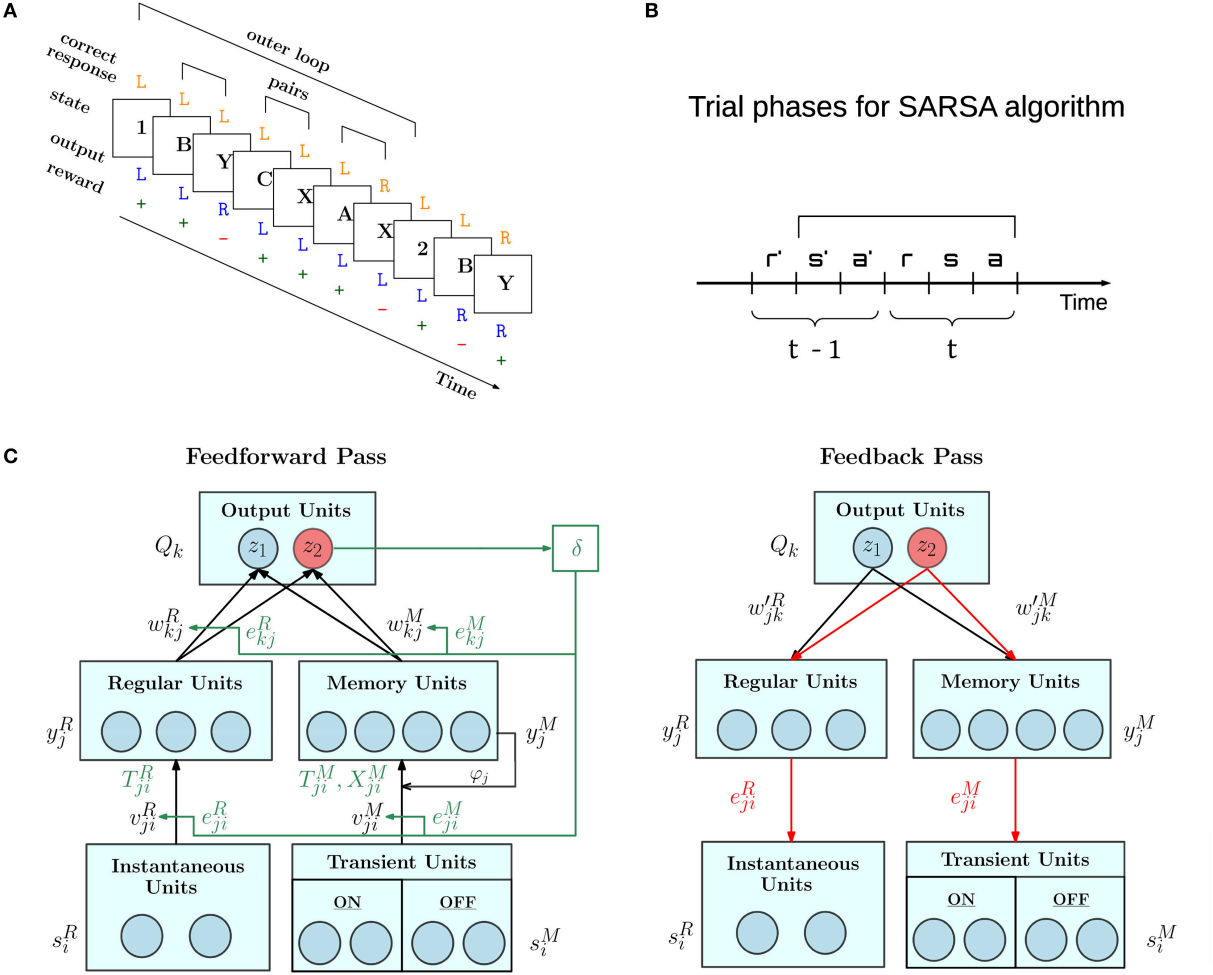
Overview of AuGMEnT network operation. **(A)** Example of trials in the 12AX task. Task symbols appear sequentially on a screen organized in outer loops, which start with a digit, either 1 or 2, followed by a random number of letter pairs (e.g., B-Y, C-X and A-X). On the presentation of each symbol, the agent must choose a Target (R) or Non-Target (L) response. If the chosen and correct responses match, the agent receives a positive reward (+), otherwise it gets a negative reward (−). Figure is adapted from Figure 1 of O'Reilly and Frank (2006). **(B)** AuGMEnT operates in discrete time steps each comprising the reception of reward (r), input of state or stimulus (s) and action taken (a). It implements the State-Action-Reward-State-Action (SARSA, in figure s'a'rsa) reinforcement learning algorithm (Sutton and Barto, 2018). In time step *t*, reward r is obtained for the previous action a' taken in time step *t* − 1. The network weights are updated once the next action a is chosen. **(C)** The AuGMEnT network is structured in three layers with different types of units. Each iteration of the learning process consists of a feedforward pass (left) and a feedback pass (right). In the feedforward pass (black lines and text), sensory information about the current stimulus in the bottom layer, is fed to regular units without memory (left branch) and units with memory (right branch) in the middle layer, whose activities in turn are weighted to compute the *Q*-values in the top layer. Based on the *Q*-values, the current action is selected (e.g., red *z*_2_). The reward obtained for the previous action is used to compute the TD error δ (green), which modifies the connection weights, that contributed to the selection of the previous action, in proportion to their eligibility traces (green lines and text). After this, temporal eligibility traces, synaptic traces and tags (in green) on the connections are updated to reflect the correlations between the current pre and post activities. Then, in the feedback pass, spatial eligibility traces (in red) are updated, attention-gated by the current action (e.g., red *z*_2_), via feedback weights.

However, in the case of more complex tasks with long trials and multiple stimuli, like 12AX (O'Reilly and Frank, 2006) depicted in Figure 1A and explained in detail in section 3.2, we find that the accumulation of information in AuGMEnT leads to a loss in performance. Hence, we ask the question whether a modified AuGMEnT model would lead to a broader applicability of attention-gated reinforcement learning. We propose a variant of the AuGMEnT network, named hybrid AuGMEnT, that introduces a range of timescales of forgetting or leakage in the memory dynamics to overcome this kind of learning limitation. We employ memory units with different decay constants so that they work on different temporal scales, while the network learns to weight their usage based on the requirements of the specific task. In our simulations, we employed just two subgroups of cells in the memory, where one half of the memory is non-leaky and the other is leaky with a uniform decay time constant; however, more generally, the hybrid AuGMEnT architecture may contain several subgroups with distinct leakage behaviors.

The paper is structured as follows. Section 2 presents the architectural and mathematical details of hybrid AuGMEnT. Section 3 describes the simulation results of the hybrid AuGMEnT network, the standard AuGMEnT network and a fully leaky control network, on two cognitive tasks, a non-hierarchical task involving sequence prediction (Cui et al., 2015) and a hierarchical task 12AX (O'Reilly and Frank, 2006). Finally, in section 4 we discuss our main achievements in comparison with state-of-the-art models and present possible future developments of the work.

## 2. Methods

### 2.1. Hybrid AuGMEnT: network architecture and operation

The network controls an agent which, in each time step *t*, receives a reward in response to the previous action, processes the next stimulus, and takes the next action (Figure 1B). In each time step, we distinguish two phases, called the feedforward pass and feedback pass (Figure 1C).

#### 2.1.1. Feedforward pass: stimulus to action selection

In AuGMEnT (Rombouts et al., 2015), information is processed through a network with three layers, as shown in the left panel of Figure 1C. Each unit of the output layer corresponds to an action. There are two pathways into the output layer: the regular *R* branch and the memory *M* branch.

The regular branch is a standard feedforward network with one hidden layer. The current stimulus $s_{i}^{R}\left(t\right)$, indexed by unit index *i* = 1, …, *S* is connected to the hidden units (called regular units) indexed by *j*, via a set of modifiable synaptic weights $v_{ji}^{R}$ yielding activity $y_{j}^{R}$:

(1) $$y_{j}^{R}(t)=\sigma \left(h_{j}^{R}\right),\;h_{j}^{R}=\sum_{i}v_{ji}^{R}s_{i}^{R}(t),$$

where σ is the sigmoidal function σ(*x*) = (1+exp(−*x*))^−1^. Input units are one-hot binary with values *S*_*i*_ ∈ {0, 1} (equal to 1 if stimulus *i* is currently presented, 0 otherwise).

The memory branch is driven by *transitions* between stimuli, instead of the stimuli themselves. The sensory input of the memory branch consists of a set of 2*S* transient units, i.e., *S* ON units $s_{l}^{+}\in \left\{0,1\right\}$, *l* = 1, 2, …, *S*, that encode the onset of each stimulus, and *S* OFF units $s_{l}^{-}\in \left\{0,1\right\}$ that encode the offset:

(2) $$\begin{array}{l} s_{l}^{+}(t)={\left[s_{l}\left(t\right)-s_{l}\left(t-1\right)\right]}_{+}\\ s_{l}^{-}(t)={\left[s_{l}\left(t-1\right)-s_{l}\left(t\right)\right]}_{+}, \end{array}$$

where the brackets signify rectification. In the following, we denote the input into the memory branch with a variable $s_{i}^{M}$ defined as the concatenation of these ON and OFF units:

(3) $$s_{i}^{M}(t)=\{\begin{array}{ll} s_{i}^{+}\left(t\right), & \text{if }i\leq S\\ s_{i-S}^{-}\left(t\right) & \text{if }i>S, \end{array}$$

The memory units in the next layer have to maintain task-relevant information through time. The transient input is transmitted via the synaptic connections $v_{ji}^{M}$ to the memory layer, where it is accumulated in the states:

(4) $$h_{j}^{M}(t)=\varphi_{j}h_{j}^{M}(t-1)+\sum_{i}v_{ji}^{M}s_{i}^{M}(t).$$

**Figure 2:**
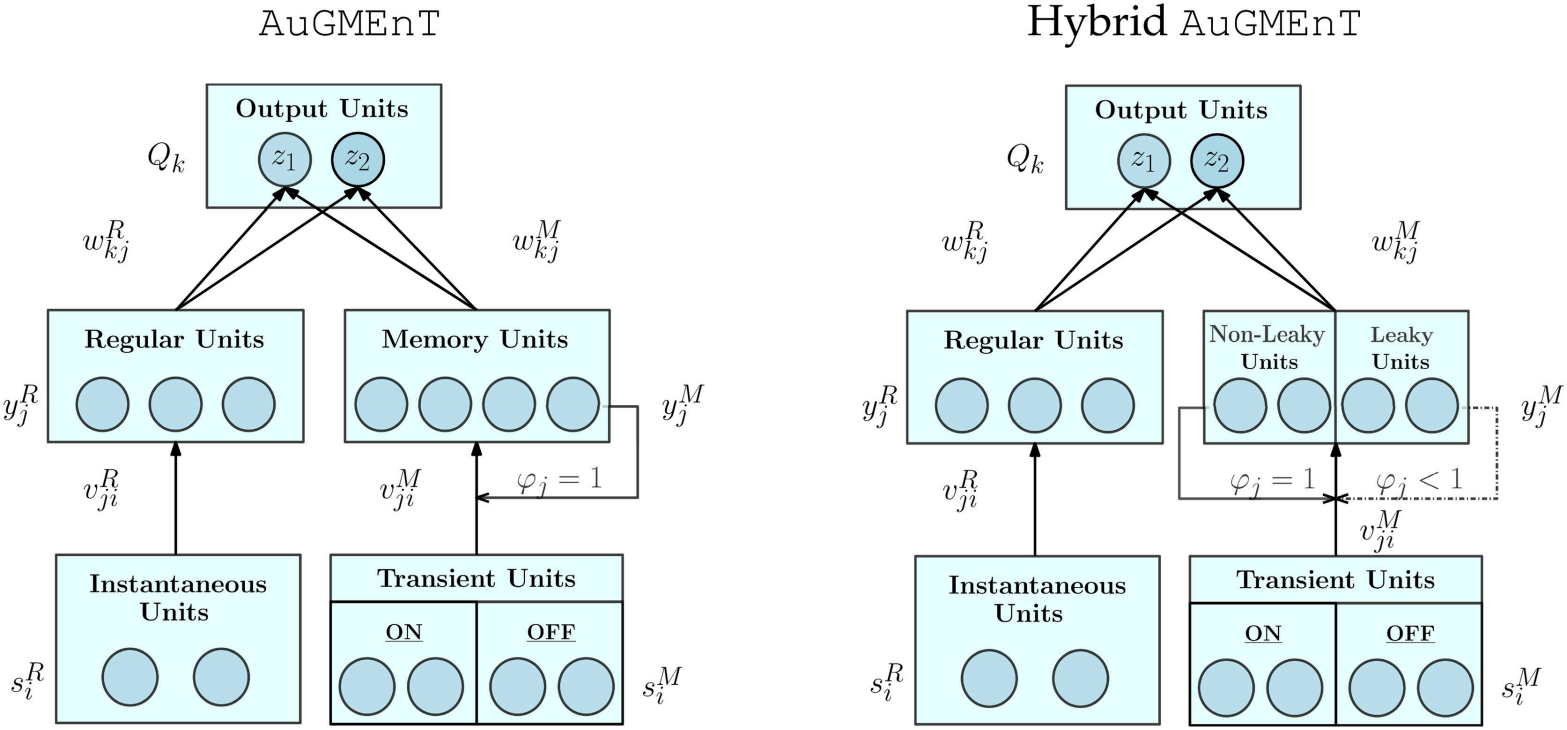
Architectures of standard AuGMEnT and hybrid AuGMEnT networks. The difference between the networks consist in their memory dynamics: the memory layer of standard AuGMEnT (left) has only conservative units with φ_*j*_ ≡ 1, while hybrid AuGMEnT (right) possesses a memory composed of both leaky φ_*j*_ < 1 and non-leaky φ_*j*_ = 1 units.

We introduce the factor φ_*j*_ ∈ [0, 1] here, as an extension to the standard AuGMEnT (Rombouts et al., 2015), to incorporate decay of the memory state $h_{j}^{M}$ over time. Setting φ_*j*_ ≡ 1 for all *j*, we obtain non-leaky memory dynamics as in the original AuGMEnT network (Rombouts et al., 2015) (Figure 2, left panel). In our hybrid AuGMEnT network, each memory cell or subgroup of memory cells may be assigned different leak co-efficients φ_*j*_ (Figure 2, right panel). In this way, the memory is composed of subpopulations of neurons that cooperate in different ways to solve a task, allowing at the same time long-time maintenance and fast decay of information in memory. In contrast to the forget gate of Long Short-Term Memory (Hochreiter and Schmidhuber, 1997) or Gated Recurrent Unit (Cho et al., 2014), our memory leak co-efficient is not trained and gated, but fixed.

The memory state $h_{j}^{M}$ leads to the activation of a memory unit:

(5) $$y_{j}^{M}(t)=\sigma \left(h_{j}^{M}(t)\right).$$

The states of the memory units are reset to 0 at the end of each trial.

Both branches converge onto the output layer. The activity of an output unit with index *k* approximates the *Q*-value of action *a* = *k* given the input **s** ≡ [*s*_*i*_], denoted as *Q*^**s**, *a*^(*t*). *Q*-values are formally defined as the future expected discounted reward conditioned on stimulus **s**(*t*) and action *a*(*t*):

(6) $$Q^{s,a}(t)=E\left[\sum_{\tau \;=\;0}^{\infty }\gamma^{\tau }r_{t+\tau +1}|s=s(t),a=a(t)\right],$$

where γ ∈ [0, 1] is a discount factor. Numerically, the vector **Q** that approximates the *Q*-values is obtained by combining linearly the hidden states from the regular and the memory branches, with synaptic weights $w_{kj}^{R}$ and $w_{kj}^{M}$:

(7) $$Q_{k}(t)=\sum_{j}w_{kj}^{R}y_{j}^{R}(t)+\sum_{j}w_{kj}^{M}y_{j}^{M}(t).$$

Finally, the *Q*-values of the different actions participate in an ϵ-greedy winner-take-all competition (Rombouts et al., 2015) to select the response of the network. With probability 1 − ϵ, the next action *a*(*t*) is the one with the maximal *Q*-value:

(8) $$a\left(t\right)={\text{argmax}}_{k}Q_{k}\left(t\right).$$

With probability ϵ, a stochastic policy is chosen with probability to take action *a* given by:

(9) $$p_{a}=\frac{\exp(g(t)Q_{a})}{\sum_{k}\exp(g(t)Q_{k})}$$

where *g*(*t*) is a weight function defined as $g\left(t\right)=1+\frac{m}{\pi }\arctan\left(\frac{t}{t^{*}}\right)$, that gradually increases in time with respect to a task-specific, fixed time scale t^*^ and a scaling factor *m*. This action selection policy is the same as that used in the original AuGMEnT (Rombouts et al., 2015), except for the weight function *g*(*t*) that we introduced, since over time this emphasizes the action with maximal *Q*-value, improving prediction stability. The time scale parameter t^*^ and the *m* factor were manually tuned to optimize convergence time. The choice of the action selection policy and these parameters is further discussed in Supplementary Material B.

#### 2.1.2. After feedforward pass: reward-based update of weights, and correlation-based update of eligibility traces, synaptic traces, and tags

AuGMEnT follows the SARSA updating scheme and updates the *Q*-values for the previous action *a*′ taken at time *t* − 1, once the action *a* at time *t* is known (see Figure 1B). *Q*-values depend on the weights via Equation (7). The temporal difference (TD) error is defined as (Wiering and Schmidhuber, 1998; Sutton and Barto, 2018):

(10) $$\delta (t)=\left(r(t)+\gamma Q_{a}(t)\right)-Q_{a^{\prime }}(t-1),$$

where *a* is the action chosen at current time *t*, and *r*(*t*) is the reward obtained for the action *a*′ taken at time *t* − 1. The TD error δ(*t*) acts as a global reinforcement signal to modify the weights of all connections as

(11) $$\begin{array}{l} v_{ji}^{R,M}(t+1)\;=v_{ji}^{R,M}(t)+\beta e_{ji}^{R,M}(t)\delta (t),\\ w_{kj}^{R,M}(t+1)=w_{kj}^{R,M}(t)+\beta e_{kj}^{R,M}(t)\delta (t), \end{array}$$

where β is a learning rate and $e_{ji}^{R,M}$ and $e_{kj}^{R,M}$ are synaptic eligibility traces, defined below; see Figure 1C. Superscript R or M denotes the regular or memory branch respectively. We use the same symbol *e*^*R, M*^ for eligibility traces at the input-to-hidden (*i* to *j*) and hidden-to-output (*j* to *k*) synapses, even though these are different, with the appropriate one clear from context and the convention for indices.

After the update of weights, a synapse from neuron *j* in the hidden layer to neuron *k* in the output layer updates its temporal eligibility trace

(12) $$\begin{array}{l} e_{kj}^{R}(t+1)=y_{j}^{R}(t)z_{k}(t)+(1-\alpha )e_{kj}^{R}(t),\\ e_{kj}^{M}(t+1)=y_{j}^{M}(t)z_{k}(t)+(1-\alpha )e_{kj}^{M}(t), \end{array}$$

where α ∈ [0, 1] is a decay parameter, *z*_*k*_ is a binary one-hot variable that indicates the winning action (equal to 1 if action *k* has been selected, 0 otherwise).

Similarly, a synapse from neuron *i* in the input layer to neuron *j* in the hidden layer sets momentary tags $T_{ji}^{R,M}$ as:

(13) $$\begin{array}{l} T_{ji}^{R}(t)=s_{i}^{R}(t)\sigma^{\prime }(h_{j}^{R}(t)),\\ T_{ji}^{M}(t)=X_{ji}^{M}(t)\sigma^{\prime }(h_{j}^{M}(t)), \end{array}$$

where $\sigma^{\prime }\left(h_{j}^{R,M}\right)$ is a non-linear function of the input potential, defined as the derivative of the gain function σ, and $X_{ji}^{M}$ is a synaptic trace (Pfister and Gerstner, 2006; Morrison et al., 2008) defined as follows:

(14) $$X_{ji}^{M}(t)=\varphi_{j}X_{ji}^{M}(t-1)+s_{i}^{M}(t).$$

Note that the tag $T_{ji}^{R,M}$ has no memory beyond one time step, i.e., it is set anew at each time step. Nevertheless, since $X_{ji}^{M}$ depends on previous times, the tag $T_{ji}^{M}$ of memory units can link across time steps. Since activities $y_{j}^{R,M}$, *z*_*k*_, $s_{i}^{R,M}$ and input potentials $h_{j}^{R,M}$ are quantities available at the synapse, a biological synapse can implement the updates of eligibility traces and tags locally. We emphasize that both eligibility traces and tags can be interpreted as 'Hebbian' correlation detectors.

In the original AuGMEnT model (Rombouts et al., 2015), all eligibility traces and tags were said to be updated in the feedback pass. Here, without changing the algorithm itself, we have conceptually shifted the update of those traces and tags that depend on the correlations of the activities, to the last step of the feedforward pass. Just as in a standard feedforward network with backpropagation of error, we rely on activities during the feedforward pass to calculate the output; therefore the algorithmic update of the weights (Roelfsema and van Ooyen, 2005; Rombouts et al., 2015) has to also rely on these feedforward activities. During the feedback pass activities of the same neurons could in principle change due to attentional gating (Moore and Armstrong, 2003; Roelfsema et al., 2010) or other feedback input. Since feedback input influences the neuronal state (Larkum et al., 1999; Larkum, 2013; Urbanczik and Senn, 2014) the activities in this second phase are different and do not carry the same information as in the feedforward phase. Thus, to increase consistency between biology and algorithm, we evaluate the correlations in the feedforward phase. An alternative could be to use multicompartmental neurons together with the assumption that feedback input arrives at distal dendrites that are only weakly coupled to proximal dendrites where most feedforward inputs arrive (Guerguiev et al., 2017) so that the state of the compartment where feedforward input arrives is only marginally influenced by feedback.

#### 2.1.3. Feedback pass: attention-gated update of eligibility traces

After action selection and the updates of weights, tags, and temporal eligibility traces in the feedforward pass, the synapses that contributed to the currently selected action update their spatial eligibility traces in an attentional feedback step. For the synapses from the input to the hidden layer, the tag $T_{ji}^{R,M}$ from Equation (13) is combined with a spatial eligibility $\underset{k}{\sum }{w^{\prime }}_{jk}^{R,M}z_{k}$ which can be interpreted as an attentional feedback signal (Rombouts et al., 2015).

(15) $$\begin{array}{l} e_{ji}^{R}(t+1)=T_{ji}^{R}\sum_{k}{w^{\prime }}_{jk}^{R}z_{k}+(1-\alpha )e_{ji}^{R}(t),\\ e_{ji}^{M}(t+1)=T_{ji}^{M}\sum_{k}{w^{\prime }}_{jk}^{M}z_{k}+(1-\alpha )e_{ji}^{M}(t), \end{array}$$

where feedback weights from the output layer to the hidden layer have been denoted as $w_{jk}^{\prime }$ and *z*_*k*_ ∈ {0, 1} is the value of output unit *k* [one-hot response vector as defined for Equation (12)].

It must be noted that the feedback synapses ${w^{\prime }}_{jk}^{R,M}$ follow the same update rule as their feedforward partner $w_{kj}^{R,M}$. Therefore, even if the initializations of the feedforward and feedback weights are different, their strengths become similar during learning.

### 2.2. Deriving the learning rule via gradient descent

For networks with one hidden layer and one-hot coding in the output, attentional feedback is equivalent to backpropagation (Roelfsema and van Ooyen, 2005; Rombouts et al., 2015). Moreover, we now show that the equations for eligibility traces, synaptic traces, and tags, along with the weight update equations reduce a TD-error-based loss function *E*:

(16) $$E=\frac{1}{2}{\left(\delta (t)\right)}^{2},$$

even in the presence of a decay factor φ < 1. Here, we specifically discuss the case of the tagging Equations (13) and (15) and the update rule (11) associated with the weight $v_{ji}^{M}$ from sensory input into memory, as these equations contain the memory decay factor φ_*j*_. Analogous update rules for weights $v_{ji}^{R}$, $w_{kj}^{M}$ and $w_{kj}^{R}$, in the hybrid AuGMEnT model are identical to existing results (Rombouts et al., 2015), and are omitted here.

Proof. We want to show that

(17) $$\Delta v_{ji}^{M}=\beta e_{ji}^{M}\delta_{t}\propto -\frac{\partial E}{\partial v_{ji}^{M}}$$

For simplicity, here we prove (17) for full temporal decay of the eligibility trace $e_{ji}^{M}$ i.e., α = 1, corresponding to TD(0) as α = 1 − γλ, so that

$$e_{ji}^{M}=T_{ji}^{M}\sum_{k}{w^{\prime }}_{jk}^{M}z_{k}=T_{ji}^{M}{w^{\prime }}_{ja^{\prime }}^{M}$$

where *a*′ is the selected action at time *t* − 1. The novel aspect of the proof is the presence of a memory decay factor φ_*j*_.

We first observe that the right-hand side of Equation (17) can be rewritten as:

$$-\frac{\partial E}{\partial v_{ji}^{M}}=-\frac{\partial E}{\partial Q_{a^{\prime }}}\frac{\partial Q_{a^{\prime }}}{\partial v_{ji}^{M}}=\delta_{t}\frac{\partial Q_{a^{\prime }}}{\partial v_{ji}^{M}}$$

Thus, it remains to show that $\frac{\partial Q_{a^{\prime }}}{\partial v_{ji}^{M}}=e_{ji}^{M}$.

Similarly to the approach used in backpropagation, we now apply the chain rule and we focus on each term separately:

$$\frac{\partial Q_{a^{\prime }}}{\partial v_{ji}^{M}}=\frac{\partial Q_{a^{\prime }}}{\partial y_{j}^{M}}\frac{\partial y_{j}^{M}}{\partial h_{j}^{M}}\frac{\partial h_{j}^{M}}{\partial v_{ji}^{M}}$$

From Equations (5) and (7), we immediately have that:

$$\frac{\partial y_{j}^{M}}{\partial h_{j}^{M}}=\sigma^{\prime }\left(h_{j}^{M}\right)\;\frac{\partial Q_{a^{\prime }}}{\partial y_{j}^{M}}=w_{a^{\prime }j}^{M}$$

We note that, in the feedback step the weight $w_{a\prime j}^{M}$ is replaced by its feedback counterpart ${w\prime }_{ja\prime }^{M}$. As discussed above, this is a valid approximation because feedforward and feedback weights become similar during learning.

Finally, for the term $\partial h_{j}^{M}/\partial v_{ji}^{M}$ starting from Equation (4) we can write:

$$\begin{array}{l} h_{j}^{M}(t)=\sum_{i}v_{ji}^{M}(t)s_{i}^{M}(t)+\sum_{\tau \;=\;t_{0}}^{t-1}\sum_{i}\varphi_{j}^{t-\tau }v_{ji}^{M}(\tau )s_{i}^{M}(\tau )\\ \;\approx \sum_{i}v_{ji}^{M}(t)\sum_{\tau \;=\;t_{0}}^{t}\varphi_{j}^{t-\tau }s_{i}^{M}(\tau ) \end{array}$$

where *t*_0_ indicates the starting time of the trial and last approximation derives from the assumption of slow learning dynamics, i.e., $v_{ij}^{M}\left(\tau \right)=v_{ij}^{M}\left(t\right)$ for *t*_0_ ≤ τ < *t*. As a consequence, we have:

$$\frac{\partial h_{j}^{M}(t-1)}{\partial v_{ji}^{M}(t-1)}\approx \sum_{\tau \;=\;t_{0}}^{t-1}\varphi_{j}^{t-\tau +1}s_{i}^{M}(\tau )=X_{ji}^{M}(t-1)$$

In conclusion, we combine the different terms and we obtain the desired result:

$$\Delta v_{ji}^{M}\propto \delta_{t}X_{ji}^{M}\sigma^{\prime }(h_{j}^{M}){w^{\prime }}_{ja^{\prime }}^{M}=\delta_{t}T_{ji}^{M}{w^{\prime }}_{ja^{\prime }}^{M}=\delta_{t}e_{ji}^{M}.$$

Thus, if the decay factor φ_*j*_ of the synaptic trace $X_{ji}^{M}$ in Equation (14) matches the decay factor of the memory unit in Equation (4), then the update rule for eligibility traces, synaptic traces and tags, and weights leads to a reduction of the TD error. However, instead of matching the two φ-s, we could merely use a unique decay factor in the input without affecting the biological plausibility of the algorithm (see Supplementary Material C). Nevertheless, we maintained the original formulation for sake of comparison with the reference AuGMEnT network.

### 2.3. Simulation and tasks

All simulation scripts were written in python (https://www.python.org), with plots rendered using the matplotlib module (http://matplotlib.org). These simulation and plotting scripts are available online at https://github.com/martin592/hybrid_AuGMEnT.

We used the parameters listed in Table 1 for our simulations. Further, for the Hybrid AuGMEnT network, we set φ_*j*_ = 1 for the first half of the memory cells and φ_*j*_ = 0.7 for the second half. To compare with the standard AuGMEnT network (Rombouts et al., 2015), we set φ_*j*_ ≡ 1 for all *j*, while for a leaky control network we set φ_*j*_ ≡ 0.7 for all *j*. In general, the leak co-efficients can be tuned to adapt the overall memory dynamics to the specific task at hand, but we did not optimize the parameter φ.

**Table 1:** Parameters for the AuGMEnT network.

**Parameter**	**Value**
β : Learning parameter	0.15
λ : Eligibility persistence	0.15
γ : Discount factor	0.9
α : Eligibility decay rate	1 − γλ
ϵ : Exploration rate	0.025
t^*^ : Softmax time scale	2000 trials
*m* : Softmax scaling factor	10

*Test dataset consists of 1, 000 random sequences, while averages are computed over 100 simulations*.

## 3. Results

AuGMEnT (Rombouts et al., 2015) includes a differentiable memory system and is trained in an RL framework with learning rules based on the joint effect of synaptic tagging, attentional feedback and neuromodulation (see Methods). Here, we study our variant of AuGMEnT, named hybrid AuGMEnT, that has an additional leak factor in a subset of memory units, and compare it to the original AuGMEnT as well as to a control network with uniform leaky memory units.

As a first step, we validated our implementations of standard and hybrid AuGMEnT networks on the Saccade-AntiSaccade (S-AS) task, used in the reference paper (Rombouts et al., 2015) (Supplementary Material D). We next simulated the networks on two other cognitive tasks with different structure and memory demands: the sequence prediction task (Cui et al., 2015) and the 12AX task (O'Reilly and Frank, 2006). In the former, the agent has to predict the final letter of a sequence depending only on its starting letter, while in the latter, the agent has to identify target pairs inside a sequence of hierarchical symbols. The S-AS task maps to a temporal XOR task (Abbott et al., 2016); thus the hidden layer is essential for the task (Minsky and Papert, 1969; Rumelhart et al., 1985). The 12AX also resembles an XOR structure, but is more complex due to an additional dimension and distractors in the inner loop (Figure S1). The complexity of the sequence prediction task is less compared to the 12AX task, and can be effectively solved by AuGMEnT. We will show that hybrid AuGMEnT performs well on both cognitive tasks, whereas standard AuGMEnT fails on the 12AX task. The parameters involving the architecture of the networks on each task are reported in Table 2. We now discuss each of the tasks in more detail.

**Table 2:** Network architecture parameters for the simulations.

**Network parameter**	**Sequence prediction task**	**12AX task**
	**(*L* = sequence length)**	
*S* : Number of sensory units	*L* − 1	8
*R* : Number of regular units	3	10
*M* : Number of memory units	8	20
*A* : Number of activity units	2	2

### 3.1. Task 1: sequence prediction

**Figure 3:**
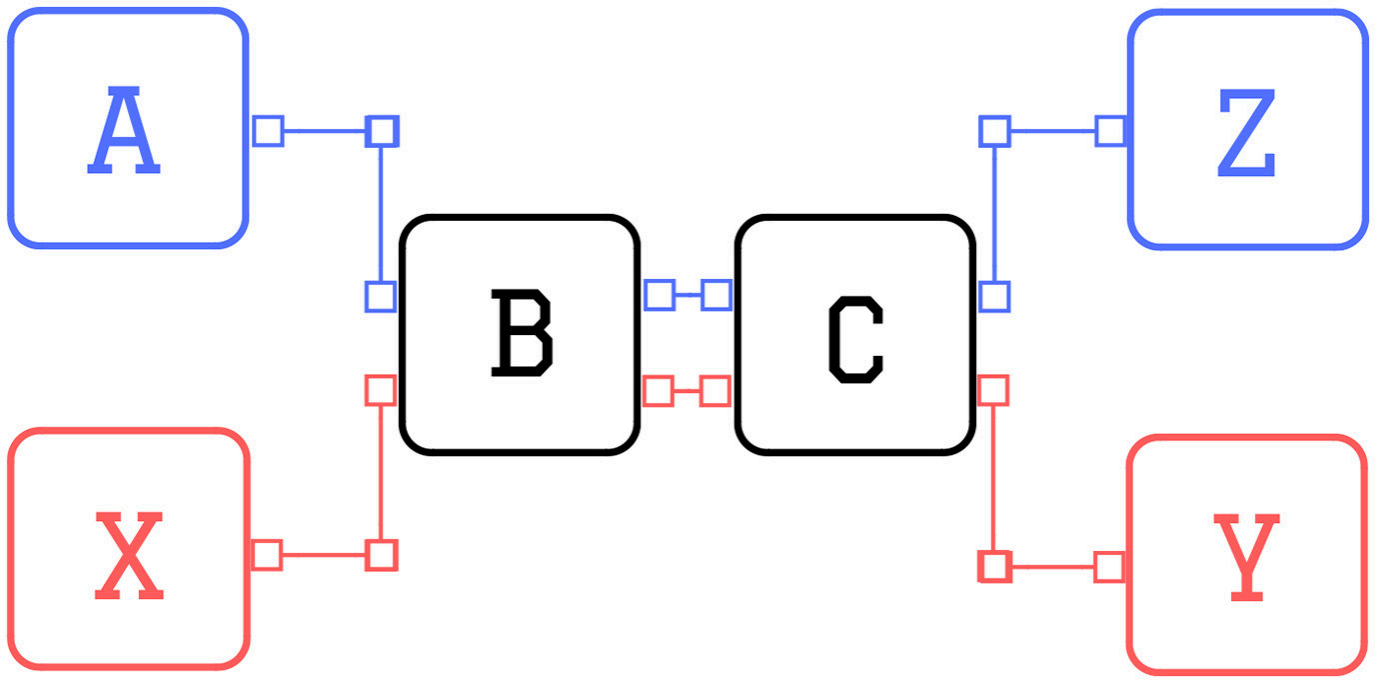
Scheme of the sequence prediction task. Scheme of sequence prediction trials with sequence length equal to 4 (i.e., 2 distractors): the two possible sequences are: A-B-C-Z (blue) or X-B-C-Y (red).

In the sequence prediction task (Cui et al., 2015), letters appear sequentially on a screen and at the end of each trial the agent has to correctly predict the last letter. Each sequence starts either with an A or with an X, which is followed by a fixed sequence of letters (e.g., B-C-D-E). The trial ends with the prediction of the final letter, which depends on the initial cue: if the sequence started with A, then the final letter has to be a Z; if the initial cue was an X, then the final letter has to be a Y. In case of correct prediction the agent receives a reward of 1 unit, otherwise it is punished with a negative reward of −1. A scheme of the task is presented in Figure 3 for sequences of four letters.

The network has to learn the task for a given sequence length, kept fixed throughout training. The agent must learn to maintain the initial cue of the sequence in memory until the end of the trial, to solve the task. At the same time, the agent has to learn to neglect the information coming from the intermediate cues (called distractors). Thus the difficulty of the task is correlated with the length of the sequence.

**Figure 4:**
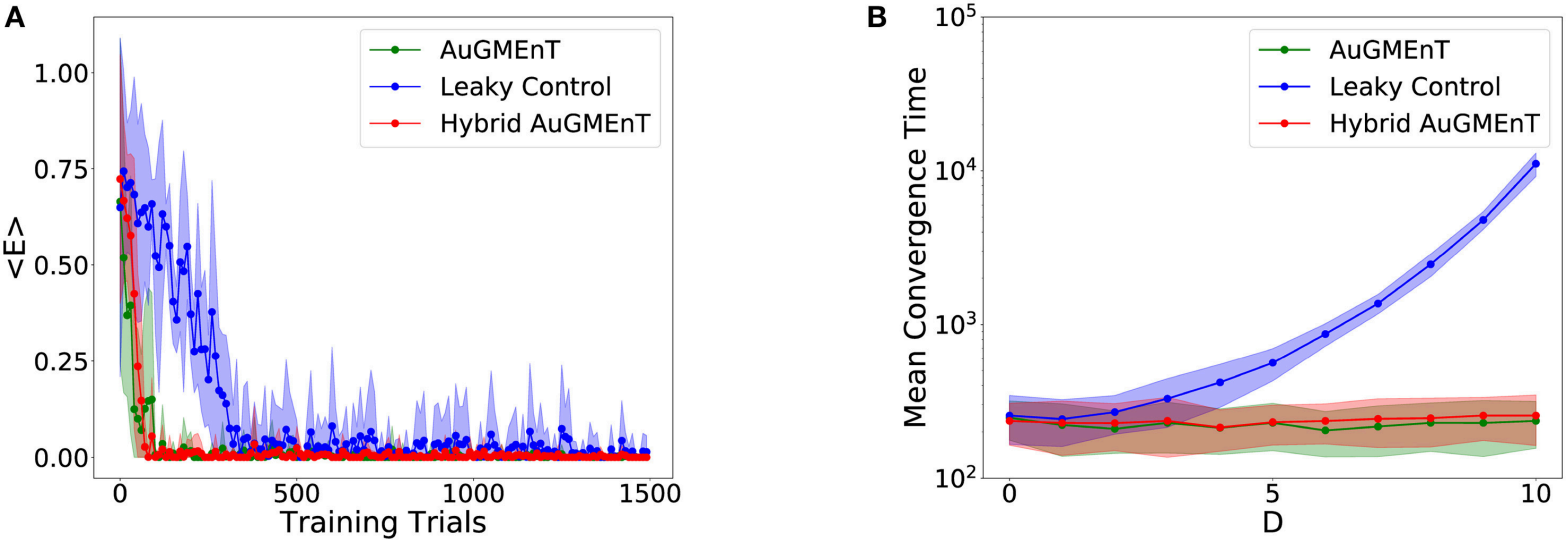
Convergence in the sequence prediction task. **(A)** Time course of error of the models on the sequence prediction task with sequences of five letters (three distractors): the mean squared TD error decays to zero for all networks, but the leaky control network (blue) is much slower than AuGMEnT (green) and Hybrid AuGMEnT (red). **(B)** Convergence time of the AuGMEnT network and its variants on the sequence prediction task with increasing number *D* of distractors, i.e., intermediate cues before final prediction.

We studied the performance of the AuGMEnT network (Rombouts et al., 2015) and our hybrid variant on the sequence prediction task. The mean trend of the TD loss function defined in Equation (16) (Figure 4A) shows that both models converge in a few hundred iterations. As a control, we also simulated a variant in which all memory units were leaky. We observed that hybrid and standard AuGMEnT networks are more efficient than the purely leaky control. This is not surprising because the key point in the sequence prediction task consists in maintaining the initial stimulus in memory—which is simpler with non-leaky memory units than with leaky ones. We notice that the hybrid model has a behavior similar to AuGMEnT.

We also analyzed the effect of the temporal length of the sequences on the network performance, by varying the number of distractors (i.e., the intermediate letters) per sequence (Figure 4B). For each sequence length, the network was retrained ab initio. We required 100 consecutive correct predictions as the criterion for convergence. We ran 100 simulations starting with different initializations for each sequence length and averaged the convergence time. Again, AuGMEnT and Hybrid AuGMEnT show good learning performance, maintaining an average of about 250 trials to convergence for sequences containing up to 10 distractors, whereas a network with purely leaky units is much slower to converge.

**Figure 5:**
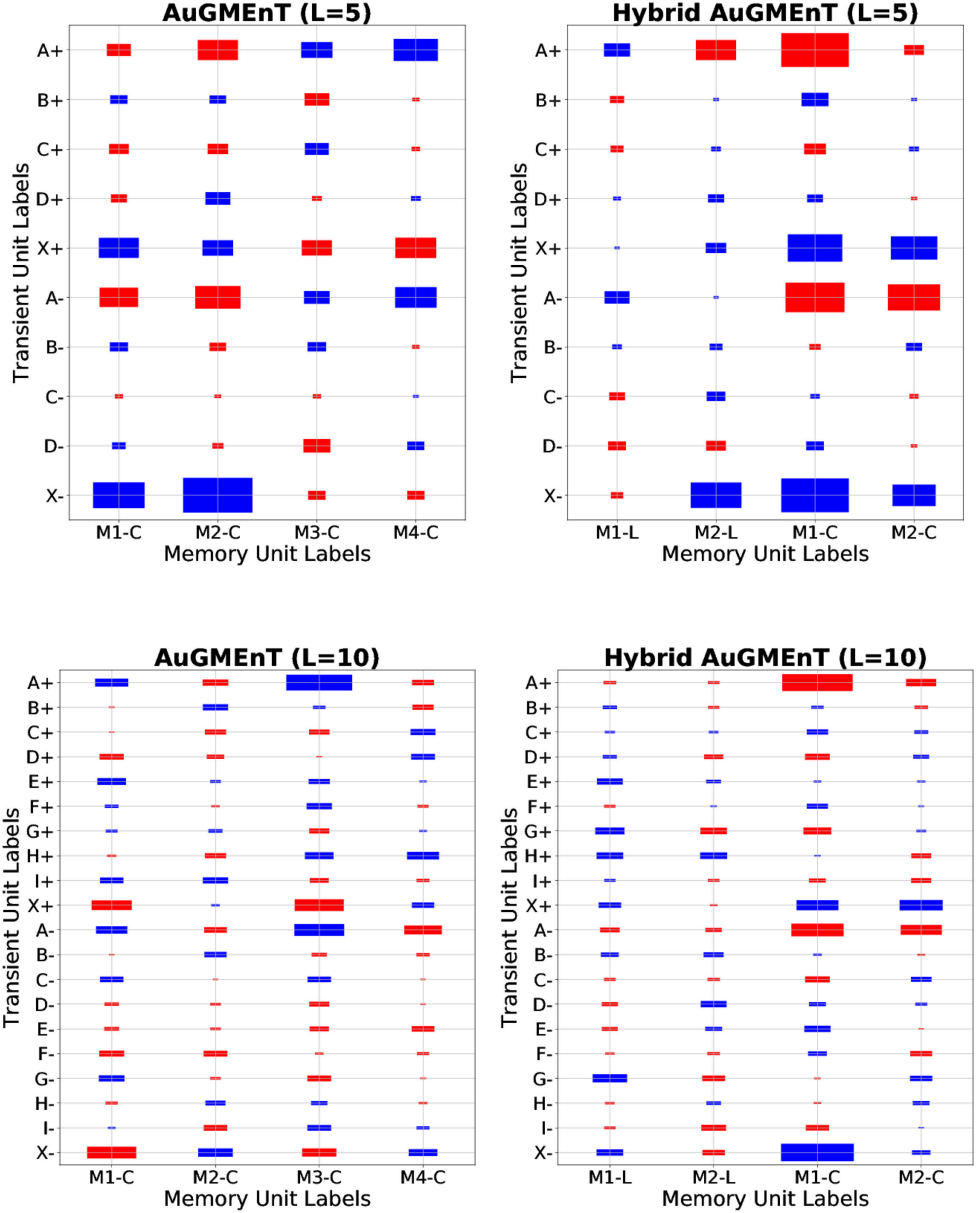
Memory weights of AuGMEnT networks in sequence prediction task for *D* = 3 and *D* = 8 distractors. Memory weight matrices (from transient units to memory units) after convergence in a representative simulation, for AuGMEnT **(left)** and Hybrid AuGMEnT **(right)** networks, on the sequence prediction task with sequences of length five (first row) and 10 (second row). Note that the first two memory units in Hybrid AuGMEnT are leaky (M1-L and M2-L), while the last ones are conservative (M1-C and M2-C). The size of each rectangle is a linear transformation of the absolute value of the weight, while the color indicates the sign (red: positive value, blue: negative value). Above: Min value = 0.01, Max value = 1.37. Below: Min value = 0.01, Max value = 1.49.

The leaky dynamics are not helpful for the sequence prediction task, because the intermediate cues are not relevant for the final model performance. Therefore, we expect the learning rule to suppress the weight values in the **V**^*M*^ matrix for distractors, and increase those of the initial A/X letter. This is confirmed by the structure of the weight matrix from transient units to memory units shown after convergence (Figure 5), in simulations of the sequence prediction task on sequences with *D* = 3 or *D* = 8 distractors. The weight values are highest in absolute value for connections from transient units representing letters A and X, for both the ON (+) and OFF (−) type. We emphasize that Hybrid AuGMEnT employs mainly the conservative (non-leaky) memory units (M1-C and M2-C) rather than the leaky ones (M1-L and M2-L) to solve the task, showing that the learning rule is able to focus updates on the connections that are most relevant for the specific task.

To confirm the better performance of the network using conservative units over leaky ones, we tested the networks on a modified task never seen during training. Specifically, the test sequences were one letter longer than training sequences and the distractors were not anymore in alphabetical order but were sampled uniformly. For instance, if the network was trained with distractors B-C-D-E, the test sequences may be A-C-D-C-B-E or X-D-B-B-B-E (the last letter remains fixed because it is the *go* signal for the network). In this way, the network experiences different forms of sequence alterations (e.g., prolongation, inversion and skipping of distractors) and we can test how the network generalizes on new versions of the problems.

Different versions of AuGMEnT are compared in the test phase by observing the mean prediction accuracy over 1, 000 test sequences (Table 3). The results show again that leaky dynamics penalize the performance on the sequence prediction task; in fact, when AuGMEnT includes conservative units (either totally or partially) the mean percentage of correct predictions is higher than 98%, otherwise the accuracy drops down to 85.8% in case of purely leaky control. However, if training is long enough to emphasize more the initial information also on leaky units, then the final test performance improves notably in both cases and the accuracy gap is greatly reduced (100% vs. 99.4%).

**Table 3:** Statistics of different versions of AuGMEnT networks tested on untrained longer-length sequences in the sequence prediction task.

**Network**	**Mean test accuracy**	
	**After convergence (%)**	**After 10, 000 training sequences (%)**
Standard AuGMEnT	98.1	100
Purely Leaky Control	85.8	99.4
Hybrid AuGMEnT	98.3	100

*Test dataset consists of 1,000 random sequences, while averages are computed over 100 simulations. The test performance is evaluated both after convergence (i.e., after few hundreds of training sequences) and after training on 10,000 trials*.

### 3.2. Task 2: 12AX

The 12AX task is a standard cognitive task used to test working memory and diagnose behavioral and cognitive deficits related to memory dysfunctions (O'Reilly and Frank, 2006; Alexander and Brown, 2015). The task involves identifying some target sequences among a group of symbols that appear on a screen.

The general procedure of the task is schematized in Figure 1A and details involving the construction of the 12AX dataset are collected in Table 4. The set of possible stimuli consists of 8 symbols: two digit cues (1 and 2), two context cues (A and B), two target cues (X and Y), and two additional distractors, a context distractor C and a distractor target Z. Each trial (or outer loop) starts with a digit cue and is followed by a random number of context-target pairs, such as A-X, B-X or B-Y. The cues are presented one by one on a screen and the agent has two possible actions for each of them: Target (R) and Non-Target (L). There are only two valid Target cases: in trials that start with digit 1, the Target is associated with the target cue X if preceded by context A (1-…-A-X); otherwise, in case of initial digit 2, the Target occurs if the target cue Y comes after context B (2-…-B-Y). The dots are inserted to stress that the target pair can occur a long time after the digit cue, as happens in the following example sequence: **1**-A-Z-B-Y-C-X-**A**-**X** (whose sequence of correct responses is L-L-L-L-L-L-L-L-**R**). The variability in the temporal length of each trial is the main challenge in solving the 12AX task. Moreover, since 1-A-X and 2-B-Y are target sequences, whereas 2-A-X and 1-B-Y are not, the task can be seen as a generalization of temporal XOR (Figure S1).

**Table 4:** The 12AX task: table of key information.

**Task feature**	**Details**
Input	8 possible stimuli: 1,2,A,B,C,X,Y,Z.
Action	Non-Target (L) or Target (R).
Target sequences	1-…-A-X or 2-…-B-Y.
	Probability of target sequence is 25%.
Training dataset	Maximum number of training trials is 1, 000, 000.
Pairs	Each trial starts with 1 or 2,
	followed by a random number (between 1 and 4) of
	pairs chosen from {A-X, A-Y, B-X, B-Y, C-X, C-Y, A-Z, B-Z, C-Z}.

The inserted pairs are determined randomly, with a probability of 50% to have pairs A-X or B-Y. As a result, combined with the probability to have either 1 or 2 as starting digit of the trial, the overall probability to have a target pair is 25%. Since the Target response R has to be associated only with an X or Y stimulus that appears in the correct sequence, the number of Non-Targets L is generally much larger, on average 8.96 Non-Targets to 1 Target. We rewarded the correct predictions of a Non-Target with 0.1 and of Targets with 1, and punished wrong predictions with a reward of −1. In effect, we balanced the positive reward approximately equally between Targets and Non-Targets based on their relative frequencies, which aids convergence.

**Figure 6:**
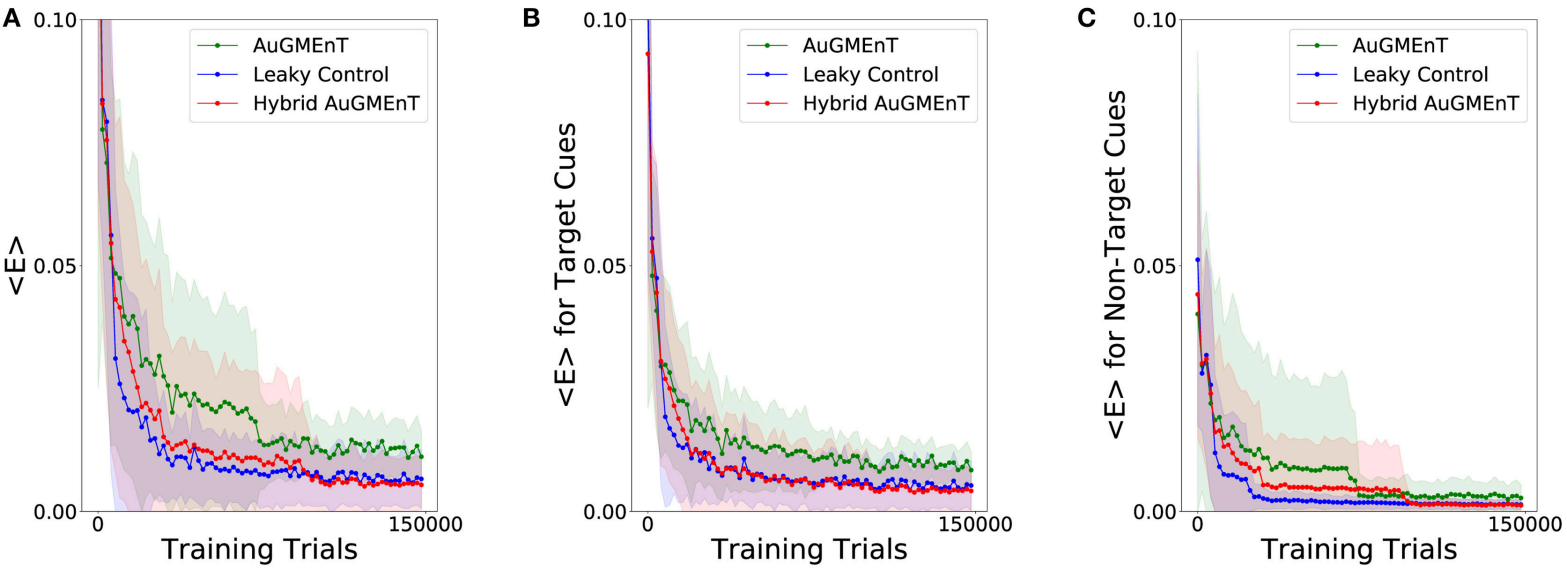
Learning convergence of the AuGMEnT variants in the 12AX task. Minimization of the TD loss function during training on the 12AX task. **(A)** All networks show a good decay of the mean squared TD error, but they seem to converge to a non-zero regime and, in particular, the base AuGMEnT network (green) maintains a higher mean squared TD error level when compared to leaky control (blue) and Hybrid AuGMEnT (red). **(B)** Mean squared TD error associated with only potential Target cues X and Y. **(C)** Mean squared TD error related to only Non-Target cues.

We simulated the Hybrid AuGMEnT network, as well as the standard AuGMEnT and the leaky control on the 12AX task, in order to see whether in this case the introduction of the leaky dynamics improves learning performance. Figure 6A shows the evolution of the mean squared TD error for the three networks. After a sharp descent, all networks converge to an error level that is non-zero, in part from ongoing action exploration and remaining part from inability to learn possibly due to memory interference. Here, hybrid AuGMEnT and leaky control saturate at a lower error value than base AuGMEnT. This difference can be attributed mainly to the errors in responding to the Target cues (Figure 6B), whereas Non-Target cues are well learned (Figure 6C). Note that, since a response is required at each step in the 12AX task, the error is also computed at each iteration—including for the more frequent and trivial Non-Target predictions—and averaged over 2,000 consecutive predictions. All networks quickly learn to recognize the Non-Target cues (1, 2, A, B, C, Z are always Non-Targets) (Figure 6C). However, hybrid AuGMEnT and leaky control learn the more complex identification of Target patterns within a trial when X or Y are presented to the network, better than base AuGMEnT (Figure 6B). The gap in the mean squared TD error between hybrid AuGMEnT and leaky control versus standard AuGMEnT is wider when only potential Target cues are considered in the mean squared TD error as in Figure 6B, than when only Non-Targets are considered as in Figure 6C.

**Figure 7:**
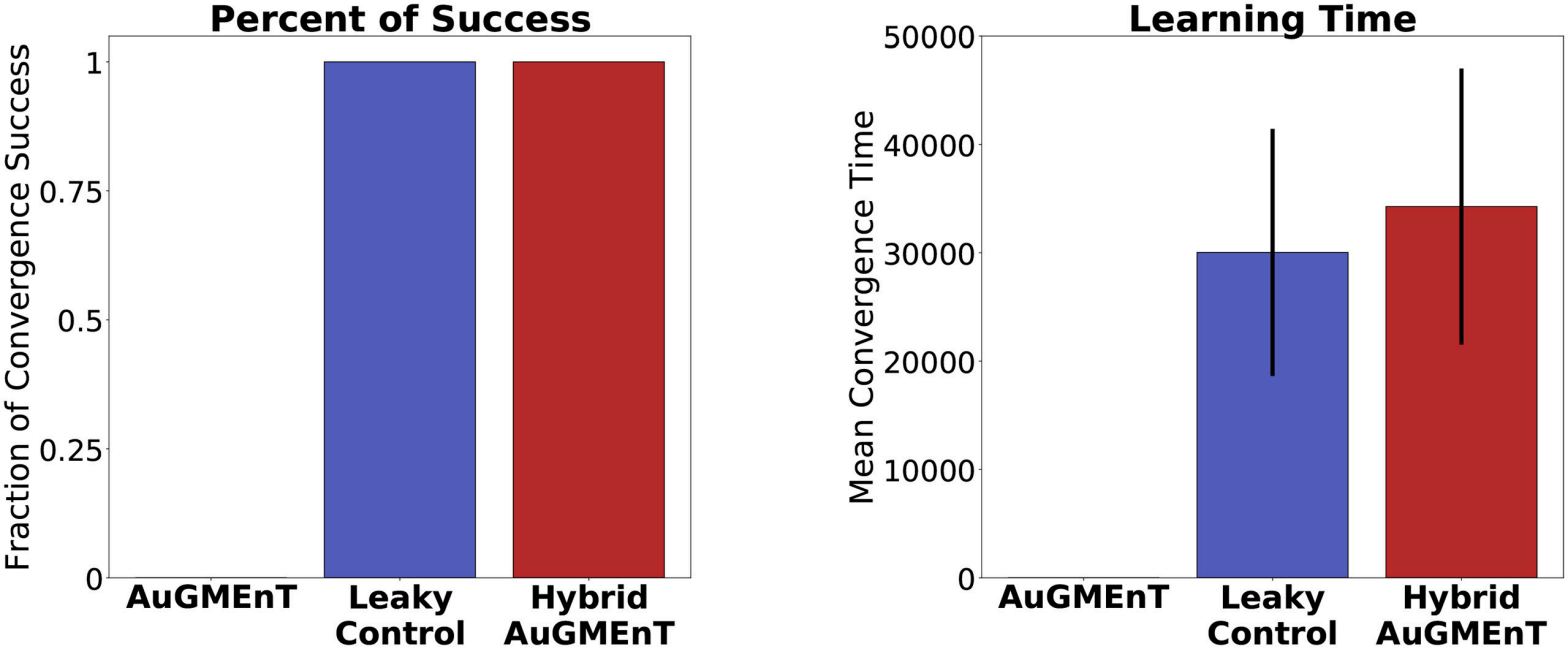
Comparative statistics of the AuGMEnT variants on performance on the 12AX task. Barplot description of the learning behavior of the three networks on the 12AX task according to the convergence criterion given by Alexander and Brown (2015). After 100 simulations, we measured the fraction of times that the model satisfies the convergence condition **(left)** and the average number of training trials needed to meet the convergence criterion **(right)**. Although training dataset consists of 1,000,000 trials, the standard AuGMEnT network never manages to satisfy the convergence criterion, while the leaky (blue) and hybrid (red) models have similar convergence performance with a learning time of about 30,000 trials.

With the convergence criterion of 1,000 consecutive correct predictions (corresponding to ~167 trials) (Alexander and Brown, 2015), standard AuGMEnT network was unable to converge (0% success), over 1,000,000 trials, in any of 100 simulations (Figure 7). However, hybrid AuGMEnT and leaky control reached 100% convergence (Figure 7), suggesting that leaky memory units are necessary for the 12AX task. The leaky control needs roughly the same time (learning time mean = 30,032.2 trials and s.d. = 11,408.9 trials) to reach convergence criterion as hybrid AuGMEnT (learning time mean = 34,263.6 trials and s.d. = 12,737.3 trials). In line with standard AuGMEnT (Rombouts et al., 2015), the memory was reset after every trial (here every outer loop), and hence the networks were not required to learn digit context switches. In Supplementary Material E, we show that leaky control and to an extent hybrid AuGMEnT also learn to switch between digit contexts, without needing the manual reset of memory. However, for the sake of comparison with the original implementation of AuGMEnT (Rombouts et al., 2015), here we default to the case with memory reset at the end of each outer loop.

Success of learning refers to the fulfillment of the convergence criterion (Alexander and Brown, 2015) and Figure 7 indicates that hybrid AuGMEnT learns well enough to reach criterion (unlike standard AuGMEnT). However, despite reaching convergence criterion after about 30,000 trials, the network may occasionally make mistakes even at the end of learning after 150, 000 trials, as indicated by the non-zero error in Figure 7. Further analysis of this result shows that the remaining errors are mainly due to our ϵ-greedy action selection policy. With a standard ϵ-greedy policy used during a separate test phase with fixed weights, about 94% of trials are successful (Table S2); however, the same network with the same synaptic weights, but a greedy policy during the test phase passes more than 98% of trials. The exact performance numbers depend on how the exploration-exploitation trade-off is implemented (see Supplementary Material B).

**Figure 8:**
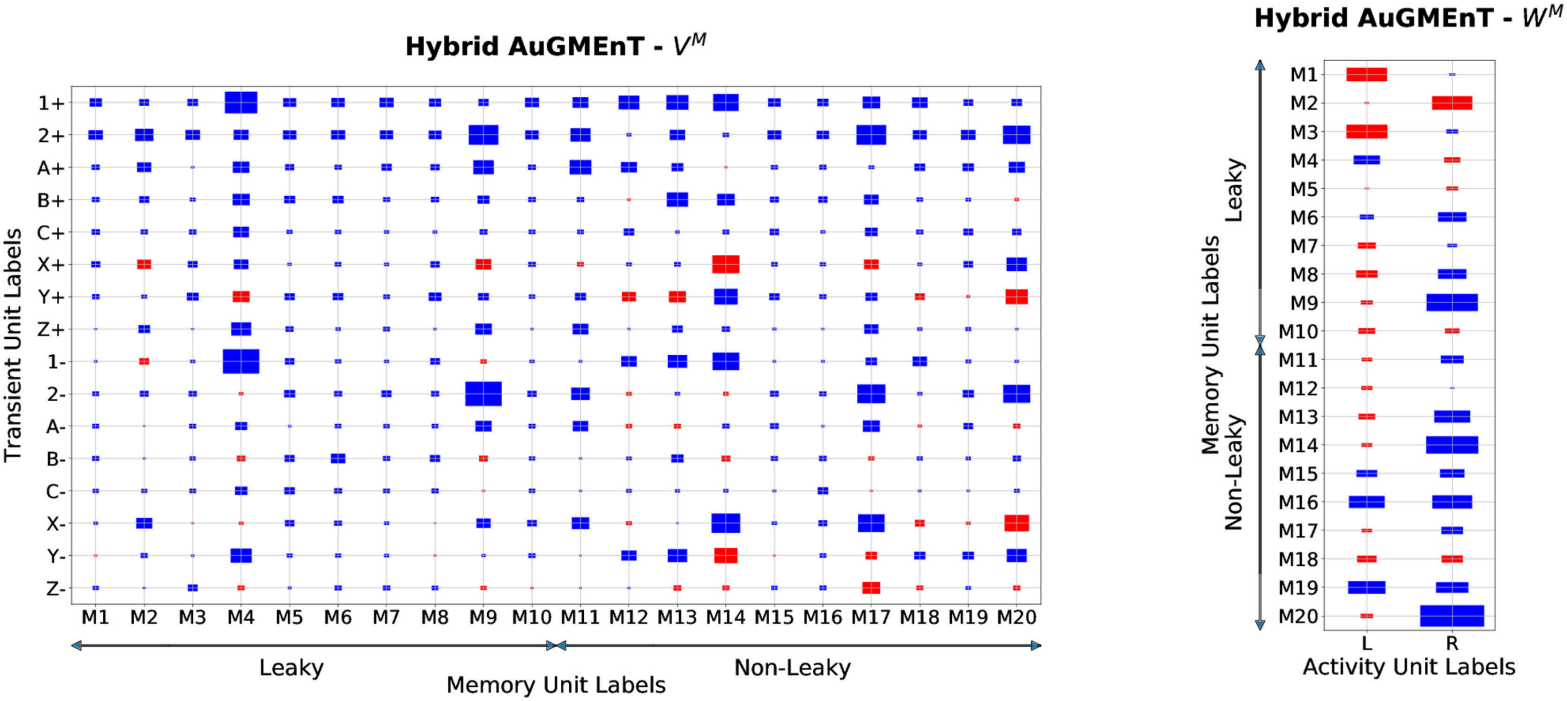
Memory weights of Hybrid AuGMEnT in the 12AX task. Plot of the weight matrices in the memory branch of hybrid AuGMEnT network after convergence in a representative simulation on the 12AX Task. The size of each rectangle is a linear transformation of the absolute value of the weight, while the color indicates the sign (red: positive value, blue: negative value). **Left:** weights from the transient stimulus into the 20 memory units (half leaky, half conservative). Min value = 0.001, Max value = 4.67. **Right:** weights from the memory cells into the output units. Min value = 0.01, Max value = 2.0.

In order to understand how the hybrid memory works on the 12AX task, we analyzed the weight structure of the connectivity matrices which belong to the memory branch of the hybrid AuGMEnT network (Figure 8). Unlike in the sequence prediction task, here the hybrid network employs both the leaky and the non-leaky memory units. The highest absolute values are found for the weights associated with 1(±) and 2(±) as well as X(±) and Y(±) (e.g., on M4, M9, M17 and M20). All memory units contribute to the definition of the activity *Q*-values (Figure 8, right panel) consistent with a distributed representation.

The memory units show an opposing behavior on activation versus on deactivation of Target cues: for instance, if X+ has strong positive weights, then X− shows negative weights (see M14, M17 or M20). In this way, the network tries to reduce the problems of memory interference between subsequent pairs by subtracting from the memory during deactivation, an amount that balances the information stored during the previous activation, effectively erasing the memory. Further, the difference in absolute value between activation and deactivation is higher in the case of the leaky cells, because the deactivation at the next iteration has to remove only a lower amount of information from the memory due to leakage. However, for the digit cues 1 and 2, the weights for activation and deactivation have typically the same sign in order to reinforce the digit signal in memory in two subsequent timesteps (e.g., on M4 and M9). More importantly, we can observe that in the leaky units, the highest weight values are generally associated to the digit information and the other cues are less represented, while in the conservative ones, the target cues are also emphasized in the memory. This means that the role of the leaky units consists mainly in the storage of the digit cue, while the conservative ones are also responsible for the storage of the information coming from the inner loops.

## 4. Discussion

The conservative dynamics of the memory in standard AuGMEnT can be a limitation for the learning ability of the model, especially in cases of complex tasks with long trials. In fact, even though the 12AX task is less complex compared to more recent RL tasks (Mnih et al., 2015), the standard AuGMEnT network fails to satisfy the convergence criterion. The introduction of the leak factor (Equation 4) in hybrid AuGMEnT leads to a network that solves the 12AX task. Hybrid AuGMEnT also does as well as standard AuGMEnT on the sequence prediction task, while the purely leaky control cannot solve this task in a reasonable time. Thus, hybrid AuGMEnT solves both tasks combining conservative and leaky memory units. Hybrid AuGMEnT can be adapted to different task structures and to different temporal scales by varying the size and the composition of the memory, for example by considering multiple subpopulations of neurons with distinct memory timescales, say in a power law distribution.

A key goal of the computational neuroscience community is to develop neural networks that are at the same time biologically plausible and able to learn complex tasks similar to humans. The embedding of memory is certainly an important step in this direction, because memory plays a central role in human learning and decision making. Our interest in the AuGMEnT network (Rombouts et al., 2015) derives from the biological plausibility of its learning and memory dynamics. In particular, the biological setting of the learning algorithm is based on synaptic tagging, attentional feedback and neuromodulation, providing a possible biological interpretation to backpropagation-like methods. Hybrid AuGMEnT inherits the biological plausibility of standard AuGMEnT. Our proposed memory mechanism is also biologically plausible with synaptic traces decaying at the memory time scale (in addition, see Supplementary Material C).

We have no convergence guarantees for our algorithm and network. While on-policy TD learning methods have convergence guarantees for fully observable Markov Decision Processes (MDPs) (Singh et al., 2000), the 12AX task is a Partially Observable Markov Decision Process (POMDP) (Monahan, 1982). Even though there are no theoretical convergence guarantees for POMDPs, there is various experimental support for solving specific POMDPs with TD learning, either using eligibility traces i.e., SARSA(λ) (Loch and Singh, 1998), or by storing observations in memory (Lin and Mitchell, 1993; McCallum, 1993; Todd et al., 2009). The memory serves to hold a history of observations, the right combination of which could represent the latent states of the underlying MDP (Sutton and Barto, 2018). Ideally, the network should learn both when and which observations (and even actions) to store in memory. While our memory does not have time-dependent gating, it does learn to weight stimuli appropriately. With gated memory and an actor-critic algorithm, the 12AX task, as well as some finite-state grammar tasks (Cleeremans and McClelland, 1991), have been learned (Todd et al., 2009). The recently proposed biologically-plausible subtractive-inhibition based gating architecture (Costa et al., 2017) could be incorporated into hybrid AuGMEnT to possibly further enhance its task repertoire.

Even apart from the issue of POMDPs, there is the issue of convergence of TD-learning for MDPs using a neural network to approximate the *Q*-value function. Here, we have used an on-policy method i.e., SARSA(λ), with the output layer being linear in the weights. There are good convergence properties for on-policy TD learning with linear (in the weights) function approximation (Tsitsiklis and Roy, 1997; Melo et al., 2008). In our network though, we also change the weights from the stimuli to the hidden units, which non-linearly affect the output. Perhaps, this can be imagined as a form of feature learning on the stimuli, these features are then combined linearly at the output (Sutton and Barto, 2018). Further, we showed that our network performs stochastic gradient descent on the squared projected TD error, projected because the network approximation might project the true TD error onto a lower-dimensional subspace (Sutton and Barto, 2018). Stochastic gradient descent on the squared projected TD error has been shown to converge with linear (Sutton et al., 2009) and non-linear (Bhatnagar et al., 2009) function approximation. Thus, even though we neither claim nor show convergence, there exists some partial and indirect theoretical support for convergence in similar architectures.

We now compare hybrid AuGMEnT with other memory-augmented networks, in order to explore different implementations of memory dynamics and possibly take inspiration for further developments on our network.

The Hierarchical Temporal Memory (HTM) network (Cui et al., 2015) presents greater flexibility in sequence learning than AuGMEnT on the simple sequence prediction task. Utilizing a complex column-based architecture and an efficient system of inner inhibitions, the HTM network is able to maintain a dual neural activity, both at column level and at unit level, that allows to have sparse representations of the input and give multi-order predictions using an unsupervised Hebbian-like learning rule. Thus, HTM has high sequence learning ability with the possibility to solve a large variety of sequence tasks, like sequence classification and anomaly detection. Nonetheless, it is unclear how the HTM network can be applied to reward-based learning, in particular to tasks like the 12AX, with variable number of inner loops.

Although the hybrid memory in the AuGMEnT network remarkably improved its convergence performance on the 12AX task, its learning efficiency is still lower than the reference Hierarchical Error Representation model (HER) (Alexander and Brown, 2015, 2016). In fact, in our simulations, hybrid AuGMEnT showed a mean convergence time of 34, 263.6 outer loops, while the average learning time of HER on the same convergence condition is around 750 outer loops. The main reason for this large gap in the learning performance resides in the gating mechanism of HER network that is specifically developed for hierarchical tasks and is used to decide at each iteration whether to store the new input or maintain the previous content in memory. Unlike HER model, the memory in AuGMEnT does not include any gating mechanism, meaning that the network does not learn when to store and recall information but the memory dynamics are entirely developed via standard weight modulation. On the other hand, the HER model is not as biologically plausible as the AuGMEnT network, because, although its hierarchical structure is inspired from the supposed organization of the prefrontal cortex, its learning scheme is artificial and based on standard backpropagation.

In addition, the recent delta-RNN network (Ororbia et al., 2017) presents interesting similarities with hybrid AuGMEnT in employing two timescales, maintaining memory via interpolation of fast and slow changing inner representations. In fact, the approach is similar to what we proposed in hybrid AuGMEnT, where the output of the memory branch is the linear combination of the activity of leaky (changing) and non-leaky (non-changing) units in the hybrid memory. The delta-RNN is a generalization of the gating mechanisms of LSTM and GRU and likely has a better learning ability than hybrid AuGMEnT, but it is less convincing in terms of biological plausibility.

The lack of a memory gating system is a great limitation for AuGMEnT variants, when compared with networks equipped with a gated memory, like HER (Alexander and Brown, 2015, 2016) or LSTM (Hochreiter and Schmidhuber, 1997; Gers et al., 2000), especially on complex tasks with high memory demand. Still, even though it cannot be properly defined as a gating system, the forgetting dynamics introduced in hybrid AuGMEnT has an effect similar to the activity of the forget gates in LSTM or GRU. However, unlike forget gates, the decay coefficients are not learnable and are not input-dependent for each memory cell.

The Hybrid AuGMEnT network could be further enhanced by adding controls on the loading, amount of leakage, and readout on the memory units, similar to input, forget, and output gates in LSTM (Hochreiter and Schmidhuber, 1997; Gers et al., 2000) and GRU (Cho et al., 2014), though only the leakage control may be most important (van der Westhuizen and Lasenby, 2018). To be specific, the value of each gate or control parameter could be set by additional units of the network that serve as a controller. In this way, the loading, leakage and output of the memory units would become stimulus- or even history-dependent. On the other hand, such a control system would make the network more complex and learning of the control variables with error backpropagation would compromise the biological plausibility of the AuGMEnT learning dynamics. However, the recently introduced subLSTM network uses subtractive inhibition in a network of excitatory and inhibitory neurons as a biologically plausible gating mechanism (Costa et al., 2017), while biologically plausible versions of backpropagation are also being developed (Lillicrap et al., 2016; Guerguiev et al., 2017; Baldi et al., 2018).

Alternatively, inspired by the hierarchical architecture of HER (Alexander and Brown, 2015), the memory in AuGMEnT could be divided into multiple levels each with its own memory dynamics: each memory level could be associated with distinct synaptic decay and leaky coefficients, learning rates, or gates, in order to cover different temporal scales and encourage level specialization. Compared with hybrid AuGMEnT, the memory would be differentiated not only in the leaky dynamics, but also in the temporal dynamics associated with attentional feedback and synaptic potentiation. Using this hierarchical structure of the memory requires additional modification of the network architecture: since the input information is separated among the memory levels, we have to introduce a system to aggregate the information. To achieve this, we could either feed the output of the hierarchical memory to the associative layer of the controller branch, or we could define a read gating system that depends on the memory content.

In the past years, the reinforcement learning community has proposed several deep RL networks, like deep Q-networks (Mnih et al., 2015) or the AlphaGo model (Chen, 2016), that combine the learning advantages of deep neural networks with reinforcement learning (Li, 2017). Thus, it may be interesting to consider a deep version of the AuGMEnT network with additional hidden layers of neurons. While conventional error backpropagation in AuGMEnT may not yield plausible synaptic plasticity rules, locality might be retained with alternative backpropagation methods (Lillicrap et al., 2016; Guerguiev et al., 2017; Baldi et al., 2018).

## Author contributions

AG, MM, and WG contributed to the conception and design of the study. MM developed and performed the simulations, and wrote the first draft of the manuscript. WG, MM, and AG revised the manuscript, and read and approved the submitted version. MM, AG and WG further revised the manuscript in accordance with the reviewers' comments and read and approved the final version.

### Conflict of interest statement

The authors declare that the research was conducted in the absence of any commercial or financial relationships that could be construed as a potential conflict of interest.
