# Supplementary material for: Multi-Timescale Memory Dynamics Extend Task Repertoire in a Reinforcement Learning Network With Attention-Gated Memory
Source: Front Comput Neurosci. 2018 Jul 12;12:50. doi: 10.3389/fncom.2018.00050 (PMC6055065; doi:10.3389/fncom.2018.00050)
Supplement: Supplementary file 1 [file Data_Sheet_1.pdf]

Multi-timescale memory dynamics extend task repertoire in a reinforcement learning network with attention-gated memory

Marco Martinolli, Wulfram Gerstner and Aditya Gilra

Supplementary Material

A) Task Structure Analogies with XOR Task

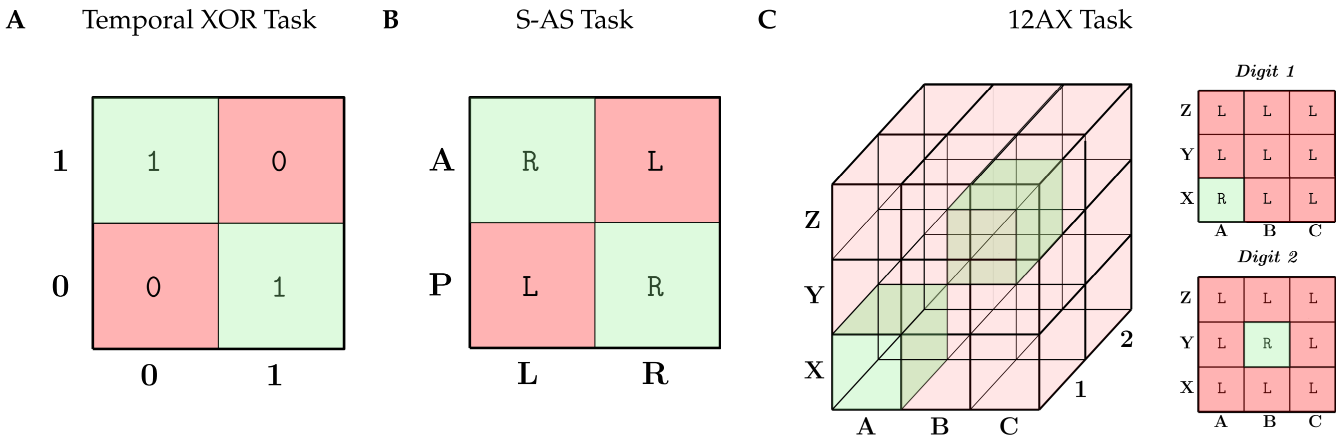

Figure S1. Spatial representation of temporal XOR, S-AS and 12AX tasks. Schematic representation of the task structures indicating for each cue combination the correct response. Temporal XOR (A) and S-AS (B) tasks have analogous input-output maps and they both have an output space that is not linearly separable. The map of 12AX task (C) is three-dimensional because its structure is based on three hierarchical levels of inputs instead of two. However, for better visualization we add at right the sections of the structure space with respect to the digit inputs that start the outer loops. The output space is still not linearly separable in 3D.

B) Comparison of Action Selection Policies

The choice of the action selection policy affects the performance of the network greatly, because it has to balance action exploration and reward exploitation properly. Here we compare the performance of hybrid `AuGMEnT` on the 12AX task under seven different action selection policies (Table S1): ϵ-greedy with softmax or uniform stochastic exploration (**P1**-**P4**), purely greedy choice (**P5**) and purely softmax policy (**P6**-**P7**). Note the use of weight function $g(t)$ introduced in Equation (9) in the softmax in policies (**P1**, **P2**, **P6**), but not in the softmax in policies (**P3**, **P7**). Policies **P1** and **P2** represent two different ways to apply weighting in time to the stochastic exploration in ϵ-greedy: the former emphasizes the Q-values more and more during training, the latter gradually reduces the frequency of response exploration. Note that **P3** is the policy used in the original `AuGMEnT` paper [Rombouts et al. 2015], to which we introduced a weight function $g(t)$ in the softmax for our default policy **P1**. The value of the ϵ parameter is set to 0.025 [Rombouts et al. 2015], for all ϵ-greedy policies for the sake of comparison.

The performance of the networks is analyzed both during training and during test (Table S2). Training statistics (columns 2 and 3) are computed with respect to the usual convergence criterion for 12AX task (i.e get $1,000$ consec-

ID	Policy Name	Stochastic Exploration	Mathematical Formulation of Stochastic Dynamics
**P1***	ϵ-greedy	Weighted softmax	$p_a = \frac{\exp(g(t)Q_a)}{\sum_k \exp(g(t)Q_k)}$ with probability ϵ, where $g(t) = 1 + \frac{m}{\pi}\arctan(\frac{t}{t^*})$
**P2**	ϵ-greedy	Weighted softmax	$p_a = \frac{\exp(Q_a)}{\sum_k \exp(Q_k)}$ with probability $\epsilon(t)$, where $\epsilon(t) = \epsilon_{max}\left(1 - \frac{2}{\pi}\arctan(\frac{t}{t^*})\right)$
**P3**†	ϵ-greedy	Softmax	$p_a = \frac{\exp(Q_a)}{\sum_k \exp(Q_k)}$ with probability ϵ
**P4**	ϵ-greedy	Uniform	$p_a = \frac{1}{K}$ $\forall a = 1:K$ with probability ϵ
**P5**	Greedy	n.a.	n.a.
**P6**	Softmax	Weighted Softmax	$p_a = \frac{\exp(g(t)Q_a)}{\sum_k \exp(g(t)Q_k)}$ where $g(t) = 1 + \frac{m}{\pi}\arctan(\frac{t}{t^*})$
**P7**	Softmax	Softmax	$p_a = \frac{\exp(Q_a)}{\sum_k \exp(Q_k)}$

Table S1. List of action selection policies used in the analysis. The exploration probability is equal in all ϵ-greedy policies for sake of comparison. $\epsilon = \epsilon_{max} = 0.025$, as per standard `AuGMEnT`[Rombouts et al. 2015]. $t^* = 2000$. $m = 10$. n.a.= not applicable. * denotes default policy used in our simulations, † denotes original policy from standard `AuGMEnT`[Rombouts et al. 2015].

utive correct predictions, corresponding to ~167 trials) on a dataset that is $100,000$ trials long. Test statistics show the prediction accuracy on $1,000$ additional trials, in which learning dynamics are stopped and action selection is purely greedy (column 4) or ϵ-greedy (column 5). The last distinction is made to highlight the effect of the stochastic exploration of ϵ-greedy on the final prediction performance. We can see that for each policy, the accuracy statistic is lower when ϵ-greedy policy is used rather than the greedy one, meaning that non-greedy exploratory actions are an additional source of errors. The limited remaining part of wrong predictions is due to rare unresolved memory interference problems occurring in particularly involved trials.

We can immediately appreciate the benefits that the weighting function $g(t)$ (or $\epsilon(t)$) has on learning success and learning time during the training phase. In fact, the weighting function causes a smooth transition towards a more deterministic action selection, in such a way that in the first phase of training, prediction exploration is encouraged to obtain a complete feedback signal on multiple scenarios, while in the second phase the Q-values are emphasized more and more to favor reward exploitation to meet the target convergence criterion.

For on-policy RL schemes, learning can converge only if the action selection policy chooses actions optimally in the limit of large time [Singh et al. 2000; Sutton and Barto 2018]. Thus we must choose the time scale t^* for exploration large enough to allow the network to explore most options, but not too large to avoid that the network never exploits. Thus, the time parameter t^* depends on the task and its convergence criterion, as well as the network. In our simulations, t^* was set to $2,000$, after manual tuning on the 12AX task. The same value of t^* was then also used for other tasks. Note that for the other tasks, the convergence criterion allows more flexibility in parameter choices. As a consequence, the influence of $g(t)$ and t^* is important mainly for the 12AX task. The value of the scaling factor m in the weighting function $g(t)$ does not substantially affect the convergence of the network, as long as it is large enough to cause a decay in exploration (Figure S2, **A**-**B**), and not too large to cause a premature reduction in exploration and thus on final prediction accuracy (Figure S2, **C**-**D**). Thus, in our simulations, we set the m factor equal to 10. Note also that if the reward is scaled by a factor x, the weighting function must be scaled by $1/x$ to obtain the same performance.

From Table S2, we see that the best overall performance in terms of convergence success, learning time and test error is with the ϵ-greedy policy with weighted softmax exploration (**P1**/**P2**). **P1** is our default policy in Results. While the greedy policy (**P5**) learns a bit faster (27.1k vs 30k trials), it has poorer convergence fraction and test scores, presumably because it does not explore during training. The weighted softmax policy **P6** has very good test

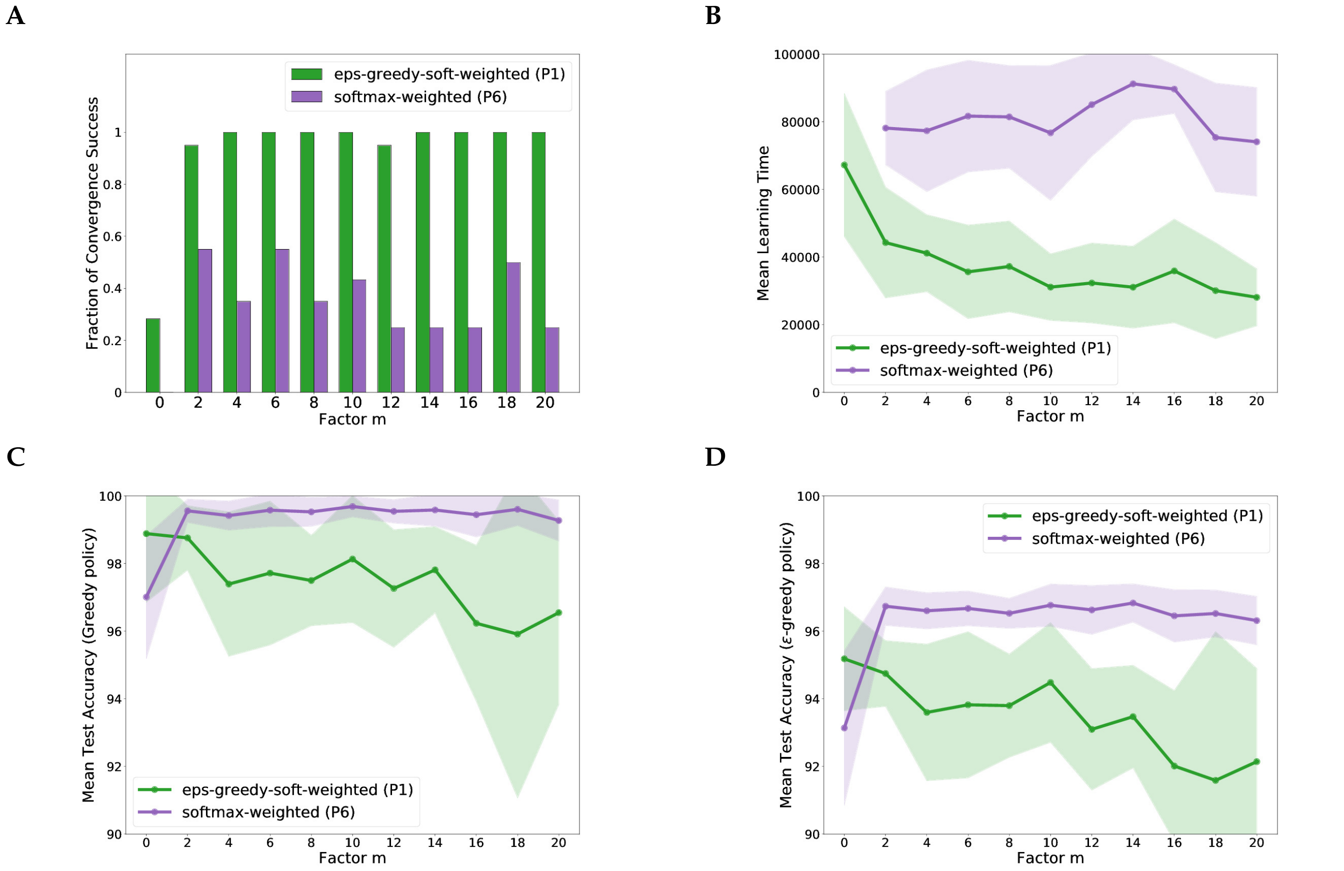

Figure S2. Variation of network performance with respect to the m scaling factor in the weighting function $g(t)$. The networks have been trained with two selection policies: ϵ-greedy with weighted soft-max (**P1**) and purely weighted softmax (**P6**), averaged over 20 simulations each for a maximum of $100,000$ training trials. **A**. The percentage of converged simulations for each value of m. **B**. The mean learning time needed to achieve the convergence criterion (for those simulations that converged). **C,D**. prediction accuracy over $1,000$ test trials after fixing the weights, using a purely greedy action selection policy (**C**) or an ϵ-greedy policy (**D**).

scores (99.7%), but takes very long to converge, both perhaps due to its assiduous exploration. The optimal policy during test might be achieved with weighted softmax **P6** if training is made much longer, i.e. with the number of training trials larger than $100,000$ that we explored here. Note also that convergence success requires $1,000$ *continuous* correct predictions unlike the test statistics, which is partly why policies **P4** and **P7** perform similar to **P1** during test, yet do not converge, though **P7** could converge with much longer training time. In Figure S3, we show the moving average of the rewards received with each selection policy in comparison with the optimal policy, i.e. with no errors made. The policies **P1** (**P2** is similar, hence not plotted) and **P5** overcome the others during training phase early on. However, as noted earlier, the purely greedy approach **P5** performs worse than **P1** in test phase, while the weighted softmax **P6** takes much longer to converge (Table S2).

	Training		Test	
**Policy ID**	**Convergence Success**	**Learning Time**	**Accuracy (greedy)**	**Accuracy (ϵ-greedy)**
**P1***	100%	31.1k (9.8k)	98.1% (1.9%)	94.5% (1.8%)
**P2**	100%	29.6k (10.5k)	98.7% (1.3%)	94.2% (1.4%)
**P3**†	28.3%	67.2k (21.1k)	98.9% (2.0%)	95.2% (1.5%)
**P4**	0%	n.a.	98.8% (1.7%)	95.1% (0.9%)
**P5**	95.0%	27.1k (8.0k)	95.9% (9.29%)	93.5% (1.1%)
**P6**	43.3%	76.7k (19.9k)	99.7% (0.3%)	96.8% (0.6%)
**P7**	0%	n.a.	97.0% (1.8%)	93.1% (2.3%)

Table S2. Training and test statistics (mean values with standard deviations in brackets) for different selection policies over 60 simulations of hybrid `AuGMEnT` on the 12AX task. Learning success and learning time are determined with respect to the convergence condition, i.e. $1,000$ consecutive correct predictions. Training phase ends on achieving convergence or after a maximum of $100,000$ trials. Mean test accuracy is computed as the percentage of $1,000$ test trials that have been solved correctly. Test statistics are presented both in case of greedy and ϵ-greedy selection policies. n.a. = not applicable. * denotes the policy used in our simulations in Results, † denotes original policy from standard `AuGMEnT` [Rombouts et al. 2015]

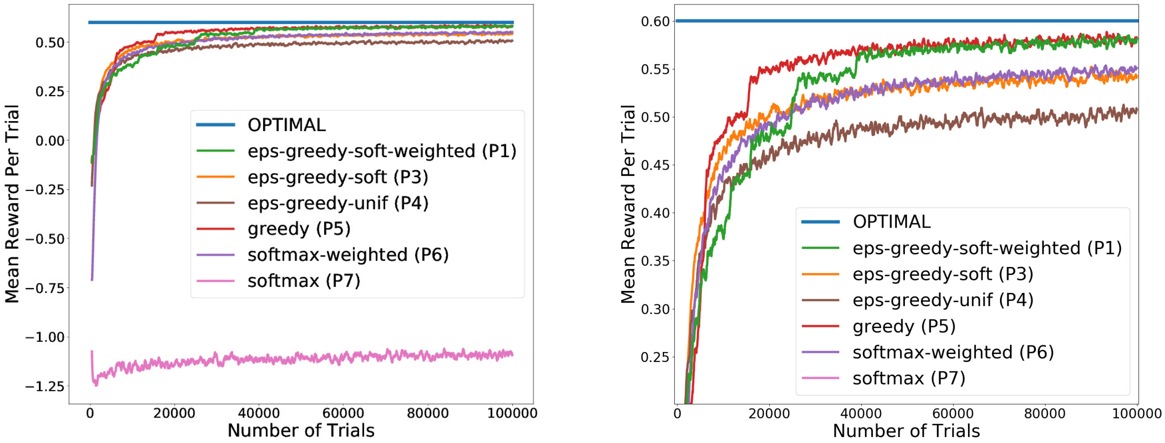

Figure S3. Comparative analysis of the reward per trial under different action selection policies. The reward is computed as the moving average of the cumulated reward in a trial, with 6 cues per trial on average. The performance is compared with the optimal policy (blue), which corresponds to perfect network behavior where no errors are committed, i.e. with average reward per trial equal to 0.6. The plot on the right is a zoomed version of the top part of the plot on the left.

C) Alternative Memory Dynamics

In our formulation of hybrid AuGMEnT, leaky dynamics are incorporated in the memory state update (Eq. (4)) and in the synaptic trace update at the end of the feedforward step (Eq. (14)). For the equivalence to backpropagation of TD error, these leaky coefficients are required to be the same φ.

Alternatively, it is possible to advance the update of the synaptic trace (Eq. (14)) before the update of the memory state. In this algorithm, the equation (4) for the memory state update can be rewritten as:

$$h_j^M(t) = \sum_i v_{ji}^M \, X_{ji}^M(t) \tag{18}$$

using the synaptic trace which is already updated with the current stimulus given at time t. The advantage of this new formulation is that it requires a unique φ parameter. Here, the synaptic trace is not independent of the memory state, but directly affects it, and must decay on the memory time scale, which is still biologically plausible.

However, the two described memory dynamics are not completely equivalent because the synaptic weights are updated at each time step. In fact, the two formulations become equivalent only under the assumption of slow learning dynamics, as shown in the following proof:

Proof. We want to show that the alternative formulation (18) is equivalent to the original memory dynamics in equation (4), under the assumption of slow learning dynamics, i.e. $v_{ji}^M(t) \simeq v_{ji}^M(t-1)$ (see *-ed approximation below).
In fact, using the definition of the synaptic trace (eq. (14)) in equation (18):

$$\begin{aligned}
h_j^M(t) &= \sum_i v_{ji}^M(t) \, X_{ji}^M(t) \\
&\overset{(14)}{=} \sum_i v_{ji}^M(t) \, \left(\varphi_j \, X_{ji}^M(t-1) + s_i^M(t)\right) \\
&= \varphi_j \sum_i v_{ji}^M(t) \, X_{ji}^M(t-1) + \sum_j v_{ji}^M(t) s_i^M(t) \\
&\overset{*}{\approx} \varphi_j \sum_i v_{ji}^M(t-1) \, X_{ji}^M(t-1) + \sum_j v_{ji}^M(t) s_i^M(t) \\
&= \varphi_j h_j^M(t-1) + \sum_i v_{ji}^M s_i^M(t)
\end{aligned}$$

□

D) Model Validation of AuGMEnT Network: The Saccade-Antisaccade Task

The Saccade-AntiSaccade Task (S-AS), presented in the AuGMEnT paper [Rombouts et al. 2015], is inspired by cognitive experiments performed on monkeys to study the memory representations of visual stimuli in the Lateral Intra-Parietal cortex (LIP). The structure of each trial covers different phases in which different cues are presented on a screen and at the end of each episode the agent has to respond accordingly in order to gain a reward. Actually, following a shaping strategy, monkeys received also an intermediate smaller reward when they learnt to fixate on the task-relevant marks at the center of the screen. The details on the procedure of the trials and the experimental results are discussed in Gottlieb and Goldberg [1999].

The agent has to look either to 'Left' (L) or to 'Right' (R) in agreement with a sequence of marks that appear on a screen at each episode. The response, corresponding to the direction of the eye movement (called saccade), depends on the combination of the location and fixation marks. The location cue is a circle displayed either at the left side (L) or at the right side (R) of the screen, while the fixation mark is a square presented at the center that

Table S3. Table of the S-AS task

Task feature	Details
Task Structure	5 phases: *start*, *fix*, *cue*, *delay*, *go*
Inputs	Fixation mark: Pro-saccade (`P`) or Anti-saccade (`A`)
	Location mark: Left (`L`) or Right (`R`)
Outputs	Eye movement: Left (`L`), Front (`F`) or Right (`R`)
Trial Types	1. `P+L=L` 2. `P+R=R`
	3. `A+L=R` 4. `A+R=L`
Training dataset	Maximum number of trials is 25,000.
	Each trial type has equal probability.
Rewards	1.5 units for correct saccade at *go*
	0.2 units for fixation of the screen in *fix*

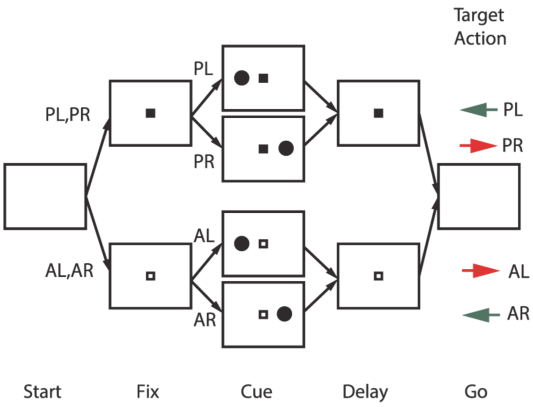

Figure S4. Structure of the Saccade-AntiSaccade task. Structure of the trials in all the possible modalities: `P-L` and `A-R` have final response `L` (green arrow), while trials `P-R` and `A-L` lead to take action `R` (red arrow). Figure taken from publication [Rombouts et al. 2015].

indicates whether the final move has to be concordant with the location cue (Prosaccade - `P`) or in the opposite direction (Antisaccade - `A`). As a consequence, there are four types of trials corresponding to the four cue combinations.

As can be seen in Figure S4, each trial is structured in five phases: a) *start*, where the screen is initially empty, b) *fix*, when the fixation mark appears c) *cue*, where the location cue is added on the screen, d) *delay*, in which the location circle disappears for two timesteps, and e) *go*, when the fixation mark vanishes as well and the agent has to give the final response to get the reward. Since the action is given at the end of the trial when the screen is completely empty, the task can be solved only if the network stores and maintains both the stimuli in memory in spite of the delay phase. In addition, the shaping strategy mentioned above is applied in the *fix* phase of the experiment, by giving an intermediate reward if the agent gazes at the fixation mark for two consecutive timesteps, to ensure that he observes the screen during the whole trial and that the *go* response is not random but consequential to the cues. So, the reward for the final response is equal to 1.5 units in case of correct response, 0 otherwise, but the intermediate reward for the shaping strategy is smaller, equal to 0.2 units. The most important details about the trial structure are summarized in Table S3. Network architecture and parameters were same as in the reference article [Rombouts et al. 2015].

The mean trend of the prediction error during training shows that learning of the S-AS task is achieved by the `AuGMEnT` network in our simulations also (Figure S5). In particular, in order to compare it with the reference performance in Rombouts et al. [2015], we applied the same convergence condition, where training on the S-AS task is considered to be successful if the accuracy for each trial type is higher than 90% in the last 50 trials. In the original paper, convergence of `AuGMEnT` is achieved 99.45% of the time, with a training of around 4,100 trials. In our simulations, the network reaches convergence every time, with a mean time of 2,063 trials (s.d.$=837.7$). The slightly better performance in our simulations could be due to minor differences in the interpretation of the task structure of

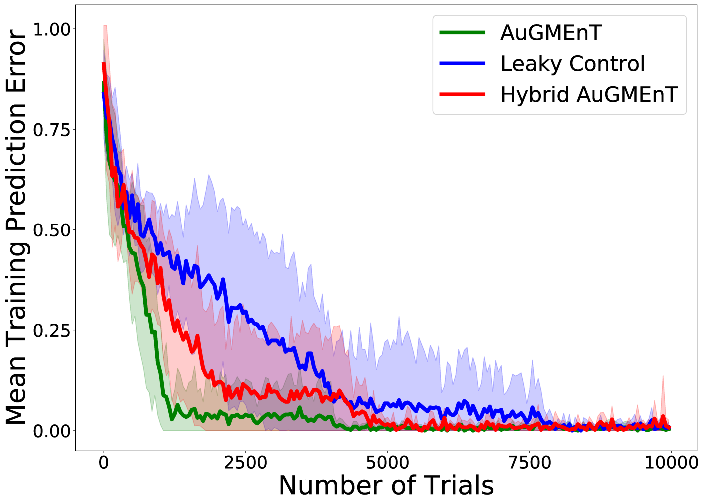

Figure S5. Validation of learning perfomance on the saccade task. Decay of the mean prediction error during training of AuGMEnT networks on the S-AS task, computed as the mean number of errors in 50 trials and averaged over 100 simulations.

S-AS or of the convergence criterion, but in any case the error plot in Figure S5 confirms a sharp decrease in the variability of the TD error after 4,000 iterations, consistent with the convergence results in the reference paper. In addition, we also show the performance of hybrid AuGMEnT and leaky control, proving that they also solve the S-AS task. However, the time to convergence is higher (especially for leaky control) because non-leaky memory is better suited to this simple task.

E) Another Benefit of Leaky Memory: Relaxation of Reset Constraint

In the original implementation of the AuGMEnT network for the S-AS task, the cumulative memory has to be reset to zero at the end of each trial [Rombouts et al. 2015]. In our Results section for the 12AX task, we reset the memory content to zero at the end of each outer loop, i.e. immediately before a digit cue, and so the network does not need to learn how to switch from context 1 to context 2, or viceversa.

However, here we show that Hybrid AuGMEnT is able to switch digit contexts to a limited extent, allowing the artificial constraint on the memory reset to be overcome. In fact, the leaky units present in the hybrid memory do not require a manual reset, because their dynamics allow continuous update and replacement of the memory content. In Table S4 we show the statistics of the simulations on two variants of the 12AX task: 12AX-triplets and continuous 12AX.

In the 12AX-triplets task, each outer loop only contains one inner loop, forming triplets of the type digit-letter-target, like 1-B-X or 2-C-Y. But, each trial is composed of a random number of up to three subsequent triplets, e.g. 2-B-Z-1-A-X. Although the memory reset is still maintained after each multi-context trial, the structure of this variant of the 12AX task allows to check whether the network is able to switch between the digit contexts or not. The results in Table S4 show that both the leaky control network and Hybrid AuGMEnT manage to solve consistently the 12AX-triplets task in short time ($\sim$ 10k training trials) and with sufficiently high test accuracy ($> 98\%$).

The continuous version of the 12AX task has the same structure as our default 12AX task (Results), but it does not include the artificial memory reset after each trial. In the continuous 12AX task, the fully leaky control network performs better than Hybrid AuGMEnT, obtaining a higher percentage of learning success (100% vs. 65%), a shorter learning time (38.3k vs. 44.9k trials) and higher test accuracy (96.2% vs. 95.2%). The conservative portion of the latter's hybrid memory seems to hinder its final performance. The leaky control network works with effectively double the amount of useful memory units for this task and without the noise introduced by the signal of the conservative units.

Here, we point out that, while the memory units were not reset across the three digit-context switches within a trial in the 12AX-triplets tasks and were also not reset across digit-contexts in the continuous version of the 12AX task, the calculation of the reward prediction error (RPE) δ did follow an outer loop structure. Since the reward for an action a' appears in the next time step, on the first time step of each digit-context, δ was taken as zero, and for a virtual time step $T+1$ after the last time step T of each digit-context, δ was computed as the difference between the reward r at time step $T+1$ and the final future-expected reward $Q_{a'}$ at time step T (assuming $Q_a(T+1) = 0$ in equation (10)). While we expect that making the calculation of RPE seamless across the outer loop will only delay convergence, further simulations are required to confirm or obtain a fully continuous working of our network to learn the continuous 12AX task.

		**Training**		**Test**
**Task**	**Model**	**Convergence Success**	**Learning Time**	**Accuracy** (greedy)
**12AX**†	Leaky Control	100%	26.7k (9.7k)	98.0% (2.1%)
	Hybrid `AuGMEnT`	100%	31.1k (9.8k)	98.1% (1.9%)
**12AX-triplets**	Leaky Control	100%	10.4k (7.4k)	98.5% (2.6%)
	Hybrid `AuGMEnT`	100%	12.5k (13.6k)	98.2% (4.8%)
**Continuous 12AX**	Leaky Control	100%	38.3k (8.0k)	96.2% (2.2%)
	Hybrid `AuGMEnT`	65%	44.9k (13.3k)	95.2% (1.9%)

Table S4. Training and test statistics (mean values with standard deviations in brackets) for different versions of the 12AX task on fully leaky control network and hybrid `AuGMEnT`. Learning success and learning time are determined with respect to the convergence condition, i.e. $1,000$ consecutive correct predictions. Mean test accuracy is computed as the percentage of $1,000$ test trials that were solved correctly (with a greedy selection policy). Statistics are averaged over the fraction of the 20 simulations that converged before a maximum of $100,000$ training trials. † denotes the default version as in Results.

References

Gottlieb, J. and Goldberg, M. E. (1999). Activity of neurons in the lateral intraparietal area of the monkey during an antisaccade task. *Nature neuroscience* 2, 906–912

Rombouts, J. O., Bohte, S. M., and Roelfsema, P. R. (2015). How attention can create synaptic tags for the learning of working memories in sequential tasks. *PLOS Computational Biology* 11, 1–34

Singh, S., Jaakkola, T., Littman, M. L., and Szepesvári, C. (2000). Convergence Results for Single-Step On-Policy Reinforcement-Learning Algorithms. *Machine Learning* 38, 287–308. doi:10.1023/A:1007678930559

Sutton, R. S. and Barto, A. G. (2018). *Reinforcement Learning: An Introduction* (MIT Press Cambridge, MA, USA), second edn.
